# Targeting LAMP2 in human cerebrospinal fluid with a combination of immunopurification and high resolution parallel reaction monitoring mass spectrometry

**DOI:** 10.1186/s12014-016-9104-2

**Published:** 2016-02-25

**Authors:** Simon Sjödin, Annika Öhrfelt, Gunnar Brinkmalm, Henrik Zetterberg, Kaj Blennow, Ann Brinkmalm

**Affiliations:** Department of Psychiatry and Neurochemistry, Institution of Neuroscience and Physiology, The Sahlgrenska Academy at the University of Gothenburg, Sahlgrenska University Hospital at Mölndal, University of Gothenburg, House V3, 431 80 Mölndal, Sweden; UCL Institute of Neurology, University College London, London, UK

**Keywords:** Alzheimer’s disease, Neurodegenerative diseases, Cerebrospinal fluid, Biomarker, LAMP2, Endo-lysosomal dysfunction, Immunoprecipitation, Parallel reaction monitoring

## Abstract

**Background:**

Alzheimer’s disease is the most common form of dementia. An increasing body of evidence suggests that endo-lysosomal dysfunction is a pathogenic mechanism of Alzheimer’s disease. Thus there is a potential for proteins involved in the normal function of endo-lysosomal vesicles to act as biomarkers of disease. Herein we focused on the lysosomal protein LAMP2 that is involved in chaperone mediated autophagy.

**Results:**

Using a combination of immunoprecipitation, digestion and nano-liquid chromatography tandem mass spectrometry we targeted and identified six tryptic LAMP2 peptides in human cerebrospinal fluid. Employing the identified proteotypic tryptic peptides a hybrid immunoprecipitation high resolution parallel reaction monitoring mass spectrometric method was developed for the relative quantitation of LAMP2. The method was evaluated in a number of experiments which defined the overall methodological as well as the analytical micro-liquid chromatography mass spectrometric intra- and inter-day variability. We identified an overall methodological peptide dependent intra-day variability of 8–16 %. The inter-day experiments showed similar results. The analytical contribution to the variation was minor with a coefficient of variation of 0.5–2.1 %, depending on the peptide. Using the developed method, with defined and limited variability, we report increased cerebrospinal fluid levels of three LAMP2 peptides in Alzheimer’s disease subjects (n = 14), as compared to non-Alzheimer’s disease controls (n = 14).

**Conclusion:**

Altered LAMP2 levels in cerebrospinal fluid may indicate endo-lysosomal dysfunction in Alzheimer’s disease. However, further studies in larger cohorts comprised of well-defined patient materials are required. We here present a tool which can be used for exploring the relevance of the level of LAMP2 as a potential measure of lysosomal dysfunction in Alzheimer’s disease or other neurodegenerative diseases.

**Electronic supplementary material:**

The online version of this article (doi:10.1186/s12014-016-9104-2) contains supplementary material, which is available to authorized users.

## Background

Alzheimer’s disease (AD) is the most common form of dementia with a prevalence increasing with the increasing age of the population [1]. At neuropathological investigation characteristic findings are extracellular plaques containing the amyloid β (Aβ) peptide [2] and intra-neuronal tangles consisting of the hyperphoshorylated and aggregated protein tau [3]. The identification of Aβ [4] instigated the development of a hypothesis stating that an imbalance in the production or clearance of the Aβ peptide is causative of the disease. This was coined the amyloid cascade hypothesis [5].

However, in a complex and heterogeneous disease such as AD that is characterized by protein aggregation, a growing body of evidence indicates that there is also a dysfunction in the endo-lysosomal system [6]. In AD patients an increased lysosomal activity and biogenesis [7–9], as well as increased rate of endocytosis with enlarged endosomes [10–13] and an extensive accumulation of autophagic vacuoles, especially in dystrophic neurites [14, 15], has been found.

There are three types of autophagy; macro- and micro-autophagy and chaperone mediated autophagy (CMA) [16]. CMA is a selective process occurring at the lysosomal membrane where KFERQ or chemically equivalent amino acid motif carrying proteins are recruited by the cytosolic chaperone protein Heat shock cognate 71 kDa protein (hsc70) to the single pass lysosomal transmembrane protein, lysosome-associated membrane glycoprotein 2 (LAMP2). The recruitment is followed by translocation and degradation of the target protein within the lysosomal lumen [17]. LAMP2 is considered to constitute the rate limiting step in CMA [17]. Furthermore, the rate of autophagy decreases with ageing [18], which in the case of CMA is due to a decline in the amount of LAMP2 [19]. This is thought to be a result of altered dynamics in the trafficking and recycling of LAMP2 from the lumen to the lysosomal membrane [20] and has potential implications in age related diseases such as AD.

A biomarker is a biomolecule that has molecular involvement in the pathological processes of the diseases it reflects [21]. There is an evident value of discovering and using biomarkers as these could aid clinical diagnosis, help in evaluating disease progress and risk or aid in monitoring treatment effects in clinical trials [22]. Cerebrospinal fluid (CSF) is in close contact with the brain and is readily accessible through lumbar puncture. Thus CSF is considered an important source for detecting and measuring soluble biomarkers reflecting disease in the central nervous system [22]. The core CSF biomarkers for AD are the 42 amino acid long Aβ peptide (Aβ_1–42_), total tau protein (T-tau), and phosphorylated tau protein (P-tau) [23]. These are part of the diagnostic criteria for research purposes developed by the International Working Group for New Research Criteria for the Diagnosis of Alzheimer’s Disease [24]. Individually these perform with good specificity and sensitivity when discriminating patients with AD from controls [25, 26]. However, when discriminating between AD and other neurodegenerative or psychiatric disorders a combination of the biomarkers or using calculated ratios between them has to be employed [23, 26]. Furthermore, doing so also has been shown to result in a prediction of conversion from mild cognitive impairment to AD with high sensitivity and specificity [27].

There is a need to find biomarkers that are prognostic, follow progression and increase our comprehension of complex neurodegenerative disorders. Given this and the pathological alterations known to occur in AD, where an increased lysosomal biogenesis [7–9] and an extensive accumulation of autophagic vacuoles [14, 15] are seen, proteins involved and associated with the endo-lysosomal system might serve as potential biomarkers reflecting this neuropathological feature of AD. Such a biomarker could hopefully add information to the established biomarkers and improve diagnostics and understanding of the disease. Recently, a number of endo-lysosomal proteins were identified at increased levels in CSF from patients with AD including LAMP2, Lysosome-associated membrane glycoprotein 1 (LAMP1), Ras related protein Rab-3 (Rab3), Ras related protein Rab-7 (Rab7), Early endosome antigen 1 (EEA1) and pro-Cathepsin L. This was accomplished using Western blotting [28].

Mass spectrometry (MS) is a powerful tool for identifying proteins in biological materials and has the potential to reflect the full complexity and diversity of the proteome. This renders it especially competent in biomarker discovery of proteins relevant in pathological conditions. Parallel reaction monitoring (PRM) is a MS method where all the fragment ions of a selected precursor ion are monitored simultaneously (i.e., in parallel) [29]. By the addition of corresponding isotope labeled peptides and the simultaneous recording of the labeled peptides’ fragment ions, quantitation can be accomplished. PRM has been enabled by the development of hybrid high resolution mass spectrometers such as the Q Exactive [30]. PRM offers highly selective and accurate measurements [29, 31] and in comparison to the alternative approach, selected reaction monitoring (SRM), PRM display similar linearity and dynamic range [29].

In this study we set out to develop a hybrid immunoprecipitation high resolution PRM-MS (IP-HR-PRM-MS) method that can be used for analyzing the level of LAMP2 in human CSF. With the developed method we report increased levels of LAMP2 peptides in CSF from individuals having an AD core biomarker profile compared to subjects with a control biomarker profile. The developed method was evaluated in a number of experiments which defined the intra- and inter-day sample variability of the method in its entirety as well as isolated to the analytical liquid chromatography (LC) tandem MS (MS/MS) detection of the analytes.

## Results

### Identification of LAMP2 in human CSF

**Fig. 1:**
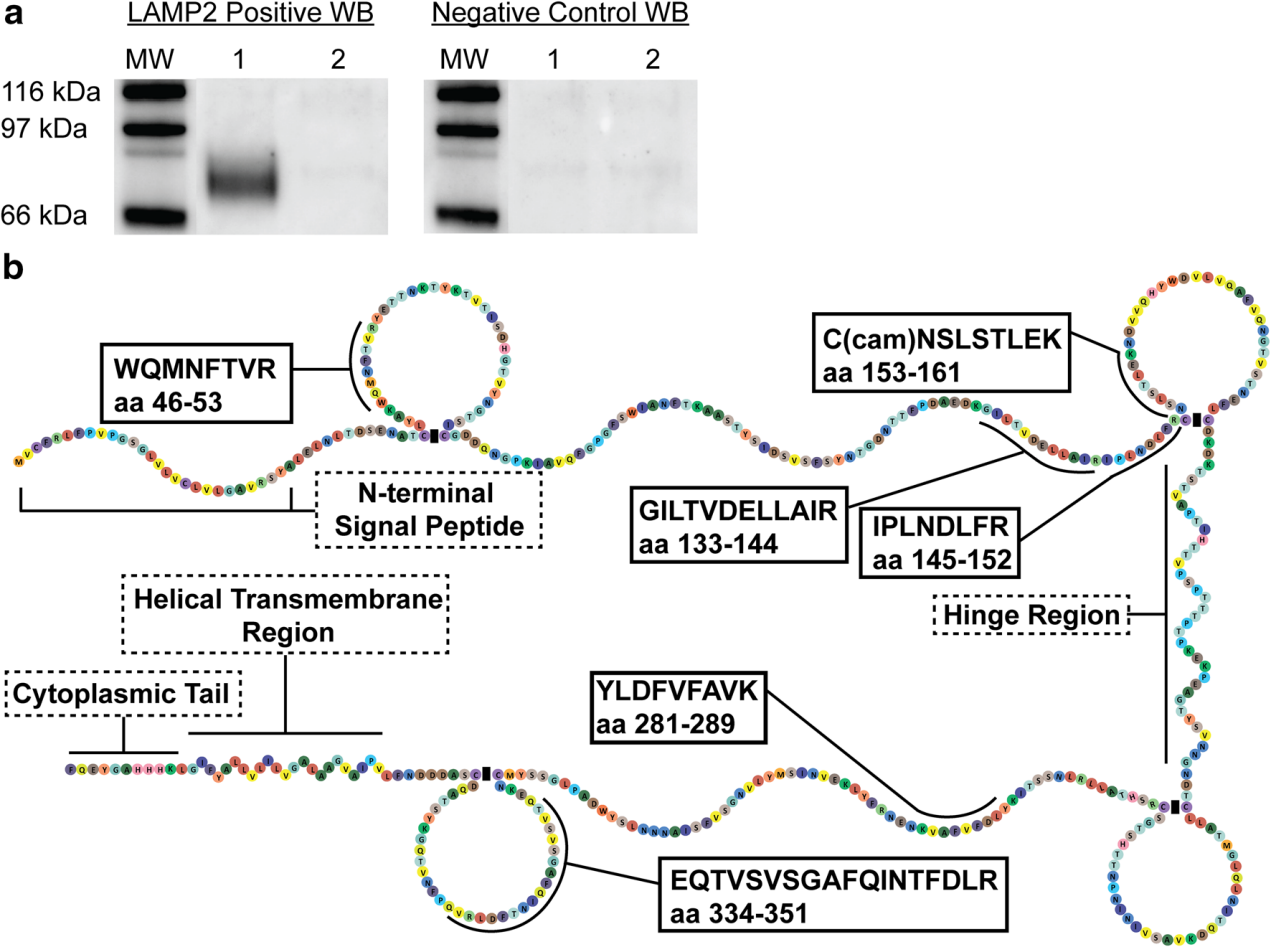
Validation of immunoprecipitation and identified LAMP2 peptides in CSF. **a** By employing Western blotting LAMP2 was detected when CSF had been immunoprecipitated with an anti-LAMP2 selective antibody, *lane 1*, and was found to migrate at approximately 80 kDa. LAMP2 was not precipitated using mouse serum IgG antibodies, *lane 2*. The detection was neither a result of unspecific binding of the secondary antibody as seen in the *right panel*. *MW* molecular weight ladder. **b** Shown is the sequence and theoretical structure of LAMP2A (UniProtKB:P13473) as indicated; signal peptide, aa 1–28; hinge region, aa 193–228; transmembrane region, aa 376–399; cytoplasmic tail, aa 400–410. Furthermore, the positions of the tryptic LAMP2 peptides identified by nano-LC–MS/MS are indicated

The selective purification of LAMP2 with IP was confirmed both by Western blotting and LC–MS/MS analysis (Fig. 1). With Western blotting LAMP2 was identified at approximately 80 kDa, Fig. 1a. Given the criteria for identification (see “Bioinformatic analysis” in “Methods” section), LAMP2 was identified in human CSF using IP, digestion with trypsin and analysis of tryptic peptides with nano-LC–MS/MS (Additional file 1: Table S1). The number of identified tryptic peptides was six when dissolving the precipitated samples with NH_4_HCO_3_ and four when using RapiGest SF (Additional file 2: Table S2). Subsequently NH_4_HCO_3_ was used to dissolve immunoprecipitated LAMP2. The best scoring peptides are presented in Table 1 and their positions along the sequence of LAMP2 are shown in Fig. 1b. As seen in the figure the peptides identified span over a large sequence range of LAMP2 (74 % sequence length coverage). Corresponding fragment ion spectra for the six identified peptides are shown in Additional file 3: Figure S1–S6. Both the Western blotting and LC–MS/MS analysis indicate that LAMP2 was selectively immunoprecipitated as no identification was made in negative controls, precipitated with an unselective antibody, by either technique. Interestingly, hsc70 (UniProtKB:P11142-2 and E9PI65) was co-immunoprecipitated and identified together with LAMP2 (Additional file 1: Table S1).

**Table 1:** Identified LAMP2 peptides by employing hybrid immunoprecipitation nano-LC–MS/MS

Sequence^a^	Peptide^b^	Mascot ion score	Mascot expect	Fragment ions^c^
WQMNFTVR	LAMP2 aa 46–53	36	4.8 × 10^−3^	–
GILTVDELLAIR	LAMP2 aa 133–144	81	6.9 × 10^−8^	y5, y6, y7, y8, y9
IPLNDLFR	LAMP2 aa 145–152	50	2.2 × 10^−4^	y4, y5, y6, y7
CNSLSTLEK	LAMP2 aa 153–161	59	2.7 × 10^−5^	y4, y5, y6, y7
YLDFVFAVK	LAMP2 aa 281–289	33	1.5 × 10^−2^	–
EQTVSVSGAFQINTFDLR	LAMP2 aa 334–351	101	1.7 × 10^−9^	y6, y7, y8, y9, y10, y11, y12, y13, y14
LGEYGFQNALIVR	BSA aa 421–433	–	–	y4, y6, y7, y8, y9, y10

The table shows the LAMP2 peptides identified in human CSF by nano-LC–MS/MS. The identification statistics values are shown for the peptides identified with highest confidence

^a^Cysteine’s were subjected to carbamidomethylation through alkylation

^b^The name of the peptides are given according to their amino acid (aa) sequence position

^c^Fragment ions used for quantitation and evaluating the HR-PRM-MS method as well as the fragment ions of the added BSA peptide

### Quantitation of LAMP2 in human CSF using IP-HR-PRM-MS

We designed a strategy for relative quantitation of LAMP2 in CSF by combining selective purification with IP, addition of stable isotope labeled standards, digestion with trypsin, and MS-based quantitation. The MS-based quantitation was performed by separation of the peptides on a Dionex UltiMate 3000 micro-LC combined with HR-PRM-MS. Initially, the performance of four selected isotope labeled peptides in the LC-HR-PRM-MS method was evaluated by the injection of three peptide mixtures from ten individual LC vials or ten times from a single vial. For each injection the sum of areas of selected fragment ions (Table 1) was normalized against the average sum of area for the group of sample replicates. Thus, the relative deviation for each injection from the group was calculated. There was no apparent difference in variation between injections from multiple LC vials or multiple times from a single vial or between the three peptide mixtures (Additional file 3: Figure S7). The coefficient of variation (CV) from injection to injection was found to vary for the LAMP2 peptides (aa 133–144, 145–152, 153–161 and 334–351) in the ranges of 3–19, 1–6, 2–8 and 24–70 %, respectively for the three peptide mixtures. For the isotope labeled bovine serum albumin (BSA) peptide aa 421–433 the CV was found to vary between 8 and 31 % (Additional file 3: Figure S8).

**Fig. 2:**
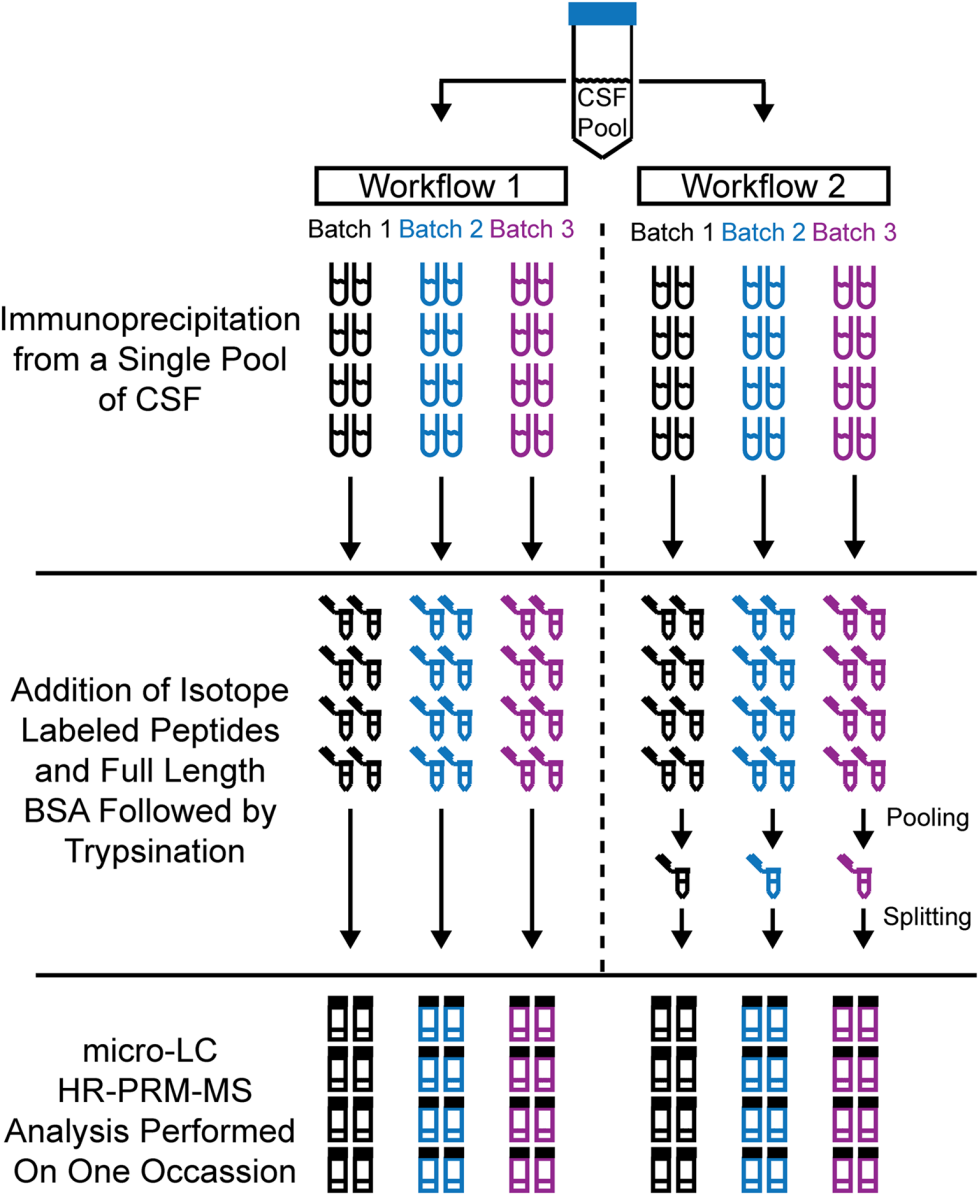
Strategy for evaluating hybrid immunoprecipitation HR-PRM-MS method variability. The variability in the IP-HR-PRM-MS method targeting selected LAMP2 peptides was evaluated by employing two methodological pathways, Workflow 1 and Workflow 2. Workflow 1 targeted the overall variability of the method whereas Workflow 2 was designed to isolate the variability in the LC-HR-PRM-MS analysis. Using a single QC pool of human CSF LAMP2 was immunoprecipitated on three separate occasions, Batch 1–3, which contained 8 + 8 replicate samples each (eight for each workflow). This enabled determination of intra- and inter-day variation. After the immunoprecipitation, isotope labeled LAMP2 peptides corresponding to the peptides previously identified using nano-LC tandem MS were added as well as full length BSA and an isotope labeled BSA peptide. The samples were then digested by trypsin. In Workflow 1 each sample was processed individually from beginning to end, thus reflecting the overall methodological variability. In Workflow 2 the eight replicate samples in each batch were pooled after trypsination. Thus, Workflow 2 was equivalent to eight injections of the same sample, for the respective batch, in the micro-LC HR-PRM-MS analysis. Although the different batches were prepared on different occasions they were all analyzed on a single occasion to minimize the influence of altering instrumental performance

**Fig. 3:**
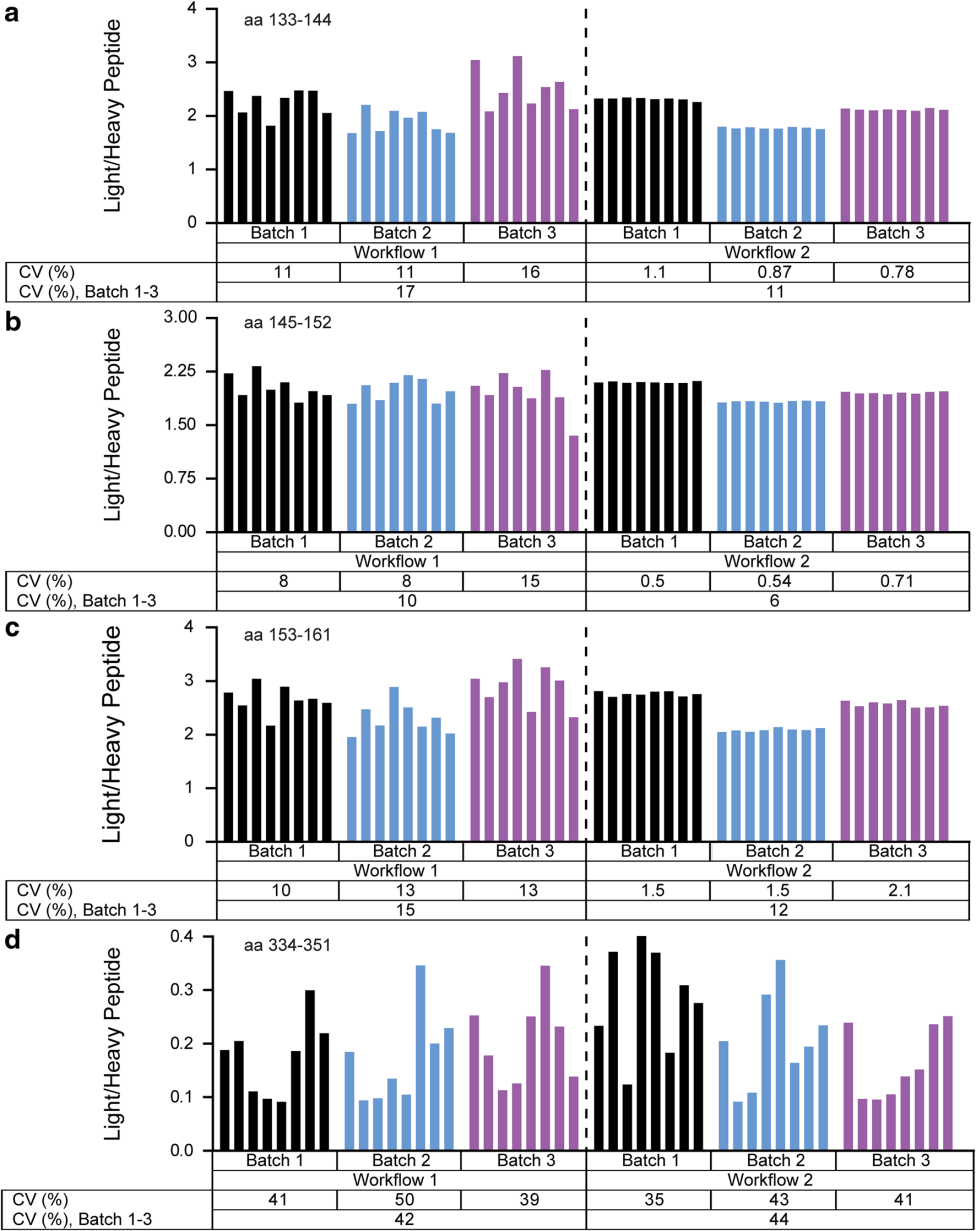
Hybrid immunoprecipitation HR-PRM-MS method variability. Using two methodological workflows (Fig. 2) the variability in the IP-HR-PRM-MS method targeting LAMP2 peptides was evaluated. Workflow 1 reflects the overall variability of the method whereas Workflow 2 isolated the variation in the micro-LC HR-PRM-MS analysis. Workflow 1 and 2 each included samples prepared on three separate occasions, Batch 1–3, each including 8 + 8 technical replicates. The different batches were analyzed on a single occasion to minimize the influence of altering instrumental performance. The workflow enabled determination of intra- and inter-day coefficients of variations (CVs), where the intraday variation was calculated for each batch and the inter-day variation calculated for the samples included in all three batches. The intra- and inter-day CVs for the workflows are shown for the LAMP2 peptides; **a** aa 133–144, **b** aa 145–152, **c** aa 153–161; and **d** aa 334–351. The *bar graphs* show the calculated ratio between the sum of the included fragment ion peak areas of the tryptic peptide against the sum of peak areas of the added isotope labeled peptide for each sample and within each batch sorted, from *left* to *right*, in order of analysis

By using two methodological workflows, the intra- and inter-day variability of the IP-HR-PRM-MS method and the analytical LC-HR-PRM-MS intra- and inter-day variability were evaluated (Fig. 2). The results of this evaluation are shown for the LAMP2 peptides in Fig. 3 and for the BSA aa 421–433 peptide in Additional file 3: Figure S9. Selected fragment ions for each peptide (Table 1) were used to calculate a ratio between the sum of fragment ion areas of the tryptic peptide against the sum of areas of the added isotope labeled peptide. The calculated ratio was used in the evaluation. The overall method variability was determined by Workflow 1 for the LAMP2 peptides (aa 133–144, 145–152, 153–161 and 334–351). The intra-day variability for the peptides was found to vary in the ranges of 11–16, 8–15, 10–13 and 39–50 %, respectively, for samples prepared on three different occasions. The corresponding inter-day CVs were 17, 10, 15 and 42 %, respectively. The intra-day CVs for the BSA peptide aa 421–433 varied between 8 and 15 % and the inter-day CV was 11 %. The analytical LC-HR-PRM-MS analysis variability was determined by Workflow 2 for the LAMP2 peptides (aa 133–144, 145–152, 153–161 and 334–351). Here the intra-day variability varied in the ranges of 0.8–1.1, 0.5–0.7, 1.5–2.1 and 35–43 % for the four peptides, respectively, between the three samples sets prepared on different occasions (Batch 1–3). The corresponding inter-day CVs were 11, 6, 12 and 44 %, respectively. The LAMP2 peptide aa 334–351 was subsequently excluded from the method due to the demonstrated high variability. The intra-day CVs varied between 1.1 and 1.7 % for the BSA peptide aa 421–433 and the inter-day CV was 5.6 %. Examples of HR-PRM total ion current chromatograms and corresponding ion fragment mass spectra are shown for the LAMP2 peptides aa 133–144, 145–152, 153–161, 334–351 and BSA aa 421–433 in Additional file 3: Figures S10–S14, respectively. Since the variation in the LC–MS method alone was limited, as determined by the above evaluation, we decided to perform a single LC-HR-PRM-MS measurement of each sample in the study below.

### Measuring the level of LAMP2 in CSF in subjects with an AD core biomarker profile

**Fig. 4:**
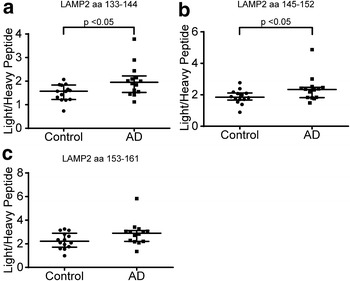
Scatterplots comparing LAMP2 peptide levels between AD and control subjects. Employing the IP-HR-PRM-MS method the level of three LAMP2 peptides were compared between a set of control (*n* = 14) and Alzheimer’s disease (AD; *n* = 14) subjects. **a** Significant higher level of the peptides **a** LAMP2 aa 133–144 (p = 0.024, 95 % CI 0.043–0.81) and **b** aa 145–152 (p = 0.039, 95 % CI 0.015–0.75) was found in the AD compared to the control group. This was not significant for the **c** aa 153–161 peptide (p = 0.10, 95 % CI −0.094 to 1.12). Shown is the calculated ratio between the sum of the included fragment ion peak areas of the tryptic peptide against the sum of peak areas of the added isotope labeled peptide for each individual subject. For each group the median and interquartile range are indicated. Groups were compared using Mann–Whitney’s U test of ranks

When employing the developed IP-HR-PRM-MS method, the levels of the three investigated tryptic LAMP2 peptides were found to be increased in subjects with an AD core biomarker profile (see “CSF samples” in “Methods” section) compared with subjects with a control biomarker profile. These increases were significant for LAMP2 aa 133–144 and 145–152 (p = 0.024, 95 % CI 0.043–0.81 and p = 0.039, 95 % CI 0.015–0.75, respectively) but not for aa 153–161 (p = 0.10, 95 % CI −0.094 to 1.12) (Fig. 4). Furthermore, a set of ten quality control (QC) CSF pool sample replicates analyzed in randomized integrated succession with the subject samples showed a variation in CV between 10 and 22 % for the LAMP2 peptides (aa 133–144, 145–152 and 153–161) and for the BSA aa 421–433 peptide (Additional file 3: Figure S15). In addition the levels of the three peptides measured in each individual in the AD group were found to be highly correlated as calculated using Spearman’s test of correlation with a Spearman’s ρ of 0.86, 0.87 and 0.93 for the peptide combinations aa 133–144/145–152, 133–144/145–152 and 145–152/153–161, respectively (Additional file 3: Figure S16A–C). The corresponding Spearman’s ρ in the control group were 0.84, 0.86 and 0.89, respectively (Additional file 3: Figure S16D–F). The slopes of all correlations were significantly different from 0 (p < 0.01). However, there was no apparent correlation between the LAMP2 peptides and the CSF core biomarkers, Aβ_1–42_, T- and P-tau with threonine phosphorylation at position 181 (P-tau_181_), neither in the AD core biomarker profile group (Additional file 3: Figure S17) nor in the control group (Additional file 3: Figure S18).

## Discussion

We identified several LAMP2 peptides in human CSF using a combination of IP, trypsination and MS. Employing the identified peptides we have developed an IP-HR-PRM-MS method that can be used to measure levels of LAMP2 in clinically relevant amounts of CSF. Using the developed method we identified higher CSF concentrations of LAMP2 tryptic peptides in subjects with an AD core biomarker profile as compared to control subjects.

The six identified LAMP2 peptides have a sequence length coverage of 74 % suggesting that a larger fragment of LAMP2 does exist in CSF. LAMP2 is produced in three spliced forms A, B and C (UniProtKB:P13473, P13473-2 and P13473-3, respectively), in which the isoform specificity is contained within the C-terminal end which remained undetected in these experiments. LAMP2 was also identified after IP using Western blotting. LAMP2 is a 45 kDa highly glycosylated protein which might explain why we detect it at 80 kDa. However, the peptides we have identified and used herein do not contain any known glycosylation sites. Furthermore, the ratios calculated for the three LAMP2 peptides aa 133–144, 145–152 and 153–161 showed a good correlation suggesting that they are a measure of the same entity and that they are produced with similar efficiency by tryptic degradation.

Next, we developed an IP-HR-PRM-MS method for quantitation of LAMP2 in CSF and determined the variation of the method. First the performance of the isotope labeled peptides in the method was investigated. Second, utilizing two methodological workflows the overall method variability and the isolated LC-HR-PRM-MS performance was defined. The latter was found to have a low contribution to the overall methodological variation. The overall methodological variation was found to be peptide dependent and to have CVs in the range of 8–16 %. We found a limited effect of preparing the samples on different days as concluded by comparing intra- with inter-day CVs. The variation identified herein using the two methodological workflows identified the non-biological contribution as all samples were prepared from a single QC CSF pool. Piehowski et al. [32] isolated the variance in different experimental parts when performing quantitative proteomics on brain tissue and found the main contributor to be dissection and homogenization (72 %) while instrumental variance only accounted for 16 %. Similarly, Addona et al. [33] identified sample preparation to be the main contributor to SRM method variation. In an IP-HR-PRM-MS method the sample preparation can be controlled for by an early addition of an internal isotope labeled standard. However, if using IP this requires that the standard carries a functional epitope and a sequence or conformation which does not impact the proteolytic processing as compared with its endogenous equivalent. Here, BSA was added as an external control of method stability to monitor the efficiency of the tryptic degradation in-between samples. Furthermore, since the same amount of BSA protein and its labeled standard was added to all samples, the LC–MS/MS stability could be monitored.

Although initially considered for inclusion in the analysis, the low repeatability of the LAMP2 peptide aa 334–351 led to its exclusion. This highlights the importance of a thorough analysis of the proteotypic peptides chosen for analysis in quantitative methods such as PRM and encourages the initial inclusion of several peptides to ensure methodological robustness and successful method development. Different peptides perform differently in PRM and other LC–MS based methods, which was indicated by the difference in variance between the peptides used in this work. This was also shown by the multicenter study performed by Addona et al. [33]. In the method described in this report the sum of areas of 4–5 fragment ions, depending on peptide, was used. The fragment ions used were selected based on having a high intensity, being reproducibly detected and having no background interference. These aspects were confirmed by manual inspection and with the assistance of an in-house developed software. A higher number of utilized fragment ions should in general improve the robustness of the method, limit the influence of interfering contaminants [33] and possible inclusion of false positive PRM peaks [34]. However, in our report individual fragment ion peak areas performed equally well as sum of areas in regard to sample variation (data not shown). Furthermore, with increasing instrumental resolution the risk for influence of contaminants decreases. In this report a MS and MS/MS resolution setting of 70,000, at 200 *m*/*z*, was used. Recently we have developed a similar PRM method targeting synaptic pathology by measuring the relative amount of the SNARE complex protein synaptosomal-associated protein 25 (SNAP-25) in human CSF and brain tissue [35, 36]. This highlights the potential of PRM methods in neurobiological biomarker discovery and development.

In a comparison between CSF from subjects with an AD core biomarker profile with subjects with a control biomarker profile (n = 14 + 14) we found a significantly increased level of LAMP2 tryptic peptides in the individuals with an AD core biomarker profile. Our findings are in agreement with Armstrong et al. [28] who also indentified increased levels of LAMP2 and a number of other endo-lysosomal proteins in a similar material of individuals. However, Armstrong et al. [28] reported a correlation between the level of LAMP2 with P-tau_181_, which we failed to replicate, potentially due to the lower number of individuals examined in our study.

An increase in the level of LAMP2 in CSF could be explained by the increased lysosomal biogenesis that occurs in neurons in AD [7–9]. In a PS1/APP mouse model Torres et al. [37] described an increased level of LAMP2 and LAMP1 in the hippocampus. The increases were associated with plaques in this region. Contrasting this, the level of LAMP2 has been suggested to decline with ageing [19]. However, both LAMP2 and hsc70 have been shown to exist at decreased levels in the substantia nigra and amygdala in subjects with Parkinson’s disease by Alvarez-Erviti et al. [38], whereas no alterations were found for LAMP2 and hsc70 in AD subjects in these two regions. We found that hsc70 was co-immunoprecipitated with LAMP2 which is interesting considering the functional interaction between these proteins in which hsc70 is responsible for the recruitment of CMA substrate KFERQ motif carrying proteins to LAMP2 [39]. The specificity of potential CSF candidate biomarkers from the endo-lysosomal system should be thoroughly investigated because of the reported involvement in several neurodegenerative diseases, such as Parkinson’s disease, Huntington’s disease, amyotrophic lateral sclerosis, frontotemporal dementia, etc., as described and reviewed elsewhere [16, 40, 41]. Also CMA, specifically, has been implicated in the degradation of proteins associated with a number of neurodegenerative diseases as exemplified in tauophaties [42], Parkinson’s disease [43–46] and Huntington’s disease [47, 48]. Furthermore, the conclusions drawn from our findings in relation to AD should be made with consideration as the individuals included herein are not clinically diagnosed.

## Conclusion

We have identified LAMP2 in human CSF using a combination of IP and MS. An IP-HR-PRM-MS method has been developed which can be used for comparing LAMP2 concentrations in clinically relevant amounts of human CSF. With the developed method which has a defined and limited methodological variability, we have measured significantly higher levels of LAMP2 peptides in a group of subjects with an AD core biomarker profile compared with a group with a control biomarker profile. Identifying new potential biomarkers might aid us in understanding complex neurodegenerative diseases. Further explorations in prospective studies, larger cohorts and/or of biomarker disease specificity are required to determine the relevance of the level of LAMP2 as a measure reflecting the neuropathology of AD or other neurodegenerative diseases. Herein we present a tool which can be used for aiding such an endeavor.

## Methods

### CSF samples

CSF samples were supplied by the clinical routine at the Clinical Neurochemistry Laboratory, The Sahlgrenska University Hospital, Mölndal, Sweden. The CSF samples were decoded. Subjects were designated as AD according to CSF AD core biomarker cut-off levels of; T-tau >400 ng/L, P-tau_181_ >80 ng/L and Aβ_1–42_ <600 ng/L. The test material included 14 subjects with an AD core biomarker profile and 14 subjects with a control biomarker profile for which the demographics are presented in Table 2. The QC CSF pool sample used for method development had a T-tau level of 167 ng/L, P-tau_181_ of 68 ng/L and Aβ_1–42_ of 400 ng/L. The study was approved by the ethics committee at the University of Gothenburg.

**Table 2:** Demographics of control and Alzheimer’s disease subjects

	Control	AD	Statistics (Mann–Whitney)
N	14	14	–
Age	70.5 (12.5)	76 (11.75)	>0.05
Female (%)	43 %	50 %	>0.05
T-tau (ng/L)	232 (97)	1035 (321.25)	<0.001
P-tau_181_ (ng/L)	38 (8.5)	91 (11.5)	<0.001
Aβ_1–42_ (ng/L)	985 (323)	450 (209.25)	<0.001

Control and Alzheimer’s disease (AD) subjects were chosen and included in the study based on having an AD or control core biomarker profile. Mann–Whitney U test of ranks were used for evaluating statistical significant differences, determined as calculated p values of <0.05. Values are presented as median (interquartile range)

### Analysis of CSF AD core biomarkers

The CSF analyses on Aβ_1–42_, T-tau and P-tau_181_ levels were performed using commercially available ELISA assays from Fujirebio [INNOTEST β-AMYLOID(1–42), INNOTEST hTau Ag and INNOTEST PHOSPHO-TAU(181P); Fujirebio Europe, Ghent, Belgium].

### Immunoprecipitation

IP from CSF was performed as described previously with minor modifications [35]. In short, 50 µL magnetic beads conjugated to anti-mouse IgG antibodies (Dynabeads M280, Invitrogen, Thermo Fisher Scientific Inc., Waltham, MA, USA), pre-washed in phosphate buffered saline (PBS) were incubated on rocking platform with 2 µg anti-LAMP2 mouse monoclonal (Abcam plc., Cambridge, UK) or mouse serum IgG antibodies (Sigma-Aldrich Co., St. Louis, MO, USA) for 1 h at room temperature. After a subsequent wash with PBS the beads were incubated on a rocking platform with Rotiblock (Carl Roth GmbH, Karlsruhe, Germany) diluted 1:10 in PBS for 1 h at room temperature. The magnetic bead–antibody complex was then washed and diluted in PBS to the original volume. The beads were added to 445 µL CSF (approximately 130 µg protein) and incubated with a final concentration of 0.2 % Triton X-100 over night at +8 °C. Using a KingFisher mL Magnetic Particle Processors system (Thermo Fisher Scientific Inc.) the beads were subsequently washed in 0.025 % Tween in PBS, PBS and finally 50 mM NH_4_HCO_3_ before being eluted in 0.5 % formic acid. The eluate was dried by vacuum centrifugation.

### Western blotting

Precipitated samples were dissolved in 1 × NuPAGE LDS sample buffer (NuPAGE, Thermo Fisher Scientific Inc.) and separated on a 4–12 % tris–glycine gel (Novex, Thermo Fisher Scientific Inc.). Proteins were transferred to a PVDF membrane (Immobilion-P, Merck KGaA, Darmstadt, Germany) which was subsequently blocked in 5 % non-fat milk in PBS with 0.05 % Tween (PBS-T). Membranes were incubated with a primary polyclonal rabbit anti-LAMP2 antibody (Santa Cruz Biotechnology, Inc., Dallas, TX, USA), diluted 1:1000 in 5 % non-fat milk in PBS-T, overnight at +8 °C. Negative controls of Western blotting were incubated without a primary antibody in 5 % non-fat milk in PBS-T. Membranes were washed in PBS-T and then incubated with a secondary anti-rabbit IgG antibody conjugated with biotin (Sigma-Aldrich Co.) diluted 1:10,000 in PBS-T, at room temperature for 1 h. After a subsequent wash in PBS-T, membranes were incubated with a streptavidin conjugated Horseradish Peroxidase (Amersham, GE Healthcare UK Ltd., Little Chalfont, UK) diluted 1:3000 in PBS-T, for 1 h at room temperature. After a final wash in PBS-T, detection of proteins were made by exposing membranes to ECL select (Amersham, GE Healthcare UK Ltd.) and by recording the chemiluminescence signal using a Fujifilm LAS-3000 camera (Fuji Photo Film Co., Ltd., Tokyo, Japan) and the Image Reader LAS-3000 v2.2 software (Fuji Photo Film Co., Ltd.). The images were finally processed by Multi Gauge v3.0 (Fuji Photo Film Co., Ltd.).

### Protein digestion for protein and peptide identification

Immunoprecipitated samples, eluted and dried, were dissolved by agitation at room temperature for 1 h after the addition of either 10 µL of 50 mM NH_4_HCO_3_ or 0.1 % RapiGest SF (Waters Co., Milford, MA, USA) in 50 mM NH_4_HCO_3_ for a qualitative comparison of recovery. 10 µL 10 mM dithiothreitol (Sigma-Aldrich Co.) in 50 mM NH_4_HCO_3_ was added to the samples which were then reduced by incubation at +90 °C for 3 min. After cooling to room temperature, 5 µL of 10 mM iodoacetamide (Sigma-Aldrich Co.) in 50 mM NH_4_HCO_3_ was added to the samples which were alkylated in the dark at room temperature for 30 min. Then, 5 µL of 5 mg/L sequencing grade modified trypsin (Promega Co., Madison, WI, USA) in 50 mM NH_4_HCO_3_ was added and the samples were incubated at +37 °C overnight (approximately 18 h). Incubation was ended by the addition of 2 µL 10 % trifluoroacetic acid after which the samples dissolved in 0.1 % RapiGest SF, were further incubated for 45 min at +37 °C. This was followed by centrifugation at +4 °C, 16,910*g* for 10 min and the resulting supernatant was transferred to 300-µL-LC vials (Sun-Sri, Thermo Fisher Scientific Inc.).

### Nano-liquid chromatography MS/MS analysis

Digested triplicate samples and corresponding immunoprecipitated negative controls were separated using a Dionex UltiMate 3000 nano-LC system (Thermo Fisher Scientific Inc.) with an Acclaim PepMap 100 nanoViper C18 trap column (length 20 mm; inner diameter 75 µm; particle size 3 µm; Thermo Fisher Scientific Inc.) and an Acclaim PepMap RSLC nanoViper C18 column (length 500 mm; inner diameter 75 µm; particle size 2 µm; Thermo Fisher Scientific Inc.). Mobile phases were; A: 0.1 % formic acid in water (v/v) and B: 0.1 % formic acid and 84 % acetonitrile in water (v/v). Separation was performed at a flow rate of 150 nL/min, at +40 °C on a gradient going from 5 to 40 % B over 50 min directly followed by an increase from 40 to 80 % B over 10 min. LC–MS/MS were acquired by a LC online connection to a hybrid quadrupole–orbitrap mass spectrometer, Q Exactive (Thermo Fisher Scientific Inc.) operating in positive ion mode with a dynamic nano-spray probe (NSI), a spray voltage of 1.7 kV and capillary transfer tube temperature of +275 °C. Full mass spectra (350–1400 *m*/*z*) were acquired at a resolution setting of 70,000 (at *m*/*z* 200), an AGC target of 1 × 10^6^ and a maximum injection time of 250 ms. This was followed by higher-energy collisional dissociation (HCD) production of fragment ions and recording of MS/MS at a resolution setting of 17,500, an AGC target of 5 × 10^4^ and a maximum injection time of 60 ms. Single micro-scans were collected with an isolation width of 2 *m*/*z* using an inclusion list of doubly and triply charged proteotypic tryptic LAMP2A, B and C peptides (UniProtKB:P13473, P13473-2 and P13473-3, respectively). When idle, data dependent top 10 MS/MS scans were collected by utilizing an intensity threshold of 1.7 × 10^4^, exclusion of unassigned, singly, and >5 + charged ions, a dynamic exclusion of 5 s and loop count of 10.

### Bioinformatic analysis

Database searches were performed using Thermo Proteome Discoverer v1.4 (Thermo Fisher Scientific Inc.) and an in-house Mascot database server v2.3.2 (Matrix Science Ltd., London, UK). Peak lists were generated using default settings. The search parameters were: database (UniProtKB_Human 131030, 88,266 sequences and 35,040,462 residues), taxonomy (all entries), enzyme (trypsin), maximum missed cleavages (1), variable modification (methionine oxidation), fixed modification (cysteine carbamidomethylation), instrument type/fragmentation type (1+ and 2+ charged b- and y-ions), peptide mass tolerance (10 ppm) and fragment mass tolerance (20 mmu). Positive protein identification was considered those proteins identified with at least two unique peptides, identified in a minimum of two replicate samples and absent in negative controls immunoprecipitated with mouse serum IgG antibodies. Positive peptide identification was considered peptides identified in a minimum of two samples, both of which received a Mascot ion score of ≥30 and a Mascot expect value of <0.05.

### Protein digestion and addition of heavy-isotope labeled peptide standards for quantitation

Immunoprecipitated dried samples were dissolved in 10 µL 50 mM NH_4_HCO_3_ containing 16 nM of the LAMP2 isotope labeled peptides aa 133–144 GILTVDELLAI[R] ([R] = ^13^C/^15^N labeled R), 145–152 IPLNDLF[R], 153–161 C[cam]NSLSTLE[K] ([cam] = cysteine carbamidomethylation; [K] = ^13^C/^15^N labeled K), 166 nM of the peptide aa 334–351 EQTVSVSGAFQINTFDL[R] and 23 nM of the BSA peptide aa 421–433 LGEYGFQNALIV[R] (HeavyPeptide FasTrack 1 crude peptides, >95 % peptide purity and >99 % isotopic enrichment, Thermo Fisher Scientific Inc.) as well as 23 nM of full length BSA protein [(UniProtKB:P02769); ≥98 % purity, Sigma-Aldrich Co.]. Samples were dissolved by agitation at room temperature for 1 h and then reduced, alkylated and digested with trypsin as described above. Trypsination was ended by the addition of 5 µL 10 % formic acid followed by centrifugation of the samples at +4 °C, 16,910*g* for 10 min after which the supernatant was transferred to 300-µL-LC vials (Sun-Sri, Thermo Fisher Scientific Inc.).

### LC-HR-PRM-MS

LC-HR-PRM-MS analyses were performed on an online LC–MS system consisting of a Dionex UltiMate 3000 standard-LC system (Thermo Fisher Scientific Inc.) coupled to a quadrupole-orbitrap mass spectrometer, Q Exactive (Thermo Fisher Scientific Inc.). 15 µL of injected samples were separated over a Hypersil GOLD HPLC C18 column (length 100 mm; inner diameter 2.1 mm; particle size 1.9 µm; Thermo Fisher Scientific Inc.) at a flow rate of 100 µL/min at +40 °C. Mobile phases were; A: 0.1 % formic acid in water (v/v) and B: 0.1 % formic acid and 84 % acetonitrile in water (v/v). A linear increase from 20 to 50 % B over 20 min was used for separation. HR-PRM MS and MS/MS acquisitions were performed with the Q Exactive operating at positive mode with a scheduled inclusion list targeting the doubly charged LAMP2 peptides aa 133–144, 145–152, 153–161, 334–351 and the BSA peptide aa 421–433 with an isolation window of 8 *m*/*z*, thus including each tryptic and isotope labeled peptide pair in a single scan. Single micro-scan MS and MS/MS acquisitions were recorded with a resolution setting of 70,000, an AGC target of 3 × 10^6^ and a maximum injection time of 300 ms. The parameters used were; a HESI-II ionization probe (Thermo Fisher Scientific Inc.) with a heater temperature of +300 °C; a spray voltage of +4.1 kV; a capillary transfer tube temperature of +320 °C; a sheath gas flow rate of 25 and an auxiliary gas flow rate of 10.

Using the isotope labeled peptides the normalized collision energy (NCE) in the HCD cell was optimized for each individual peptide used in the PRM analysis. This was accomplished by direct infusion of respective peptide solution. A short method employing acquisitions of both intact peptide ion spectra and fragment ion spectra having different NCE settings was utilized. The best NCE setting could then be determined by calculating the respective fragment ion-to-precursor ion-area ratio or simply by manually inspecting the quality of the fragment spectra.

### IP-HR-PRM-MS method variability

Using the described HR-PRM-MS analysis configuration, the analysis behavior of the isotope labeled peptides was investigated by the injection of three separate mixtures of the these peptides diluted in 50 mM NH_4_HCO_3_ to a final concentration of 3 nM LAMP2 aa 133–144, 145–152, and 153–161; 33 nM of LAMP2 aa 334–351; and 7 nM of BSA aa 421–433. Of the isotope labeled peptide mixtures 15 µL were injected in replicates of ten from multiple LC vials or ten times from a single vial. The variability of the IP-HR-PRM-MS method was evaluated using an approach divided in two pathways, Workflow 1 and Workflow 2, described schematically in Fig. 2. In short, Workflow 1 was used for evaluating the overall variability of the total IP-HR-PRM-MS method whereas Workflow 2 targeted the variability solely in the LC-HR-PRM-MS analysis. Initially, LAMP2 was immunoprecipitated from the QC sample of which 8 + 8 replicate samples were prepared for each workflow on three separate occasions denominated Batch 1–3. In Workflow 1 each sample was prepared and analyzed individually. In Workflow 2 the eight replicate samples prepared on the same occasion were pooled before the LC-HR-PRM-MS analysis, thus being equivalent to multiple injections of the same sample. The samples immunoprecipitated and digested with trypsin on three different occasions were analyzed by LC-HR-PRM-MS on a single occasion in a randomized integrated succession. For this reason the analysis was only to a limited extent compromised by varying instrumental performance.

### Quantitative analyses

Using selected fragment ions, see Table 1, produced from tryptic and isotope labeled peptide pairs, PinPoint v1.4 (Thermo Fisher Scientific Inc.) was employed for determining fragment ion peak areas. This was done using a MS accuracy setting of 10 ppm centered at 0, a MS/MS accuracy of 10 ppm and the isolation mode set to MS/MS with an isolation width setting of 9 u. The peaks were detected using a chromatographic peak width setting of 0.5 min and a minimum signal threshold of 1. A possible retention time alignment error of 2 min was allowed. The complete peak area was determined after using four points of smoothing. The detected fragment ion peaks were manually inspected for accuracy and absence of interferences from other compounds than the peptide of interest. The exported fragment ion peak areas were processed using an in-house developed software, which facilitated further inspection of possible interferences. Then, for each peptide, the sum of the included fragment ion peak areas were calculated followed by calculation of the ratio between the sum of fragment ion peak areas of the tryptic peptide against the sum of areas of the added isotope labeled peptide. The calculated values used in this report are presented in Additional file 4: Table S3.

### Statistics

Statistical analyses were performed using GraphPad Prism v6.04 (GraphPad Software, Inc., La Jolla, CA, USA). Shapiro–Wilk test for normality was used in combination with distribution statistics to evaluate the normality in the distribution, where a test statistics of a p value ≥0.05 suggests normality. Non-parametric Mann–Whitney U test of ranks was used for comparisons between groups where statistical significance was determined as p values <0.05 and a 95 % CI not comprising 0. Spearman’s test of correlation was used to evaluate the correlation between the measured levels of LAMP2 peptides and between the peptides and the AD core biomarkers. The correlation was evaluated using Spearman’s ρ and was considered to have a slope significantly different from 0 when p < 0.01.

## Additional files

10.1186/s12014-016-9104-2 Identified proteins following IP of LAMP2 in human CSF.
10.1186/s12014-016-9104-2 Identified peptides following IP of LAMP2 in human CSF.
10.1186/s12014-016-9104-2  Supporting Information. Table legends for **Tables S1**–**S3** and Supplementary **Figures S1**–**S18**.
10.1186/s12014-016-9104-2 Quantitative data produced by the HR-PRM-MS method and raw-data processing.

## Abbreviations

Aβ: amyloid β
Aβ_1–42_: 42 amino acid long amyloid β peptide
AD: Alzheimer’s disease
BSA: bovine serum albumin
CMA: chaperone mediated autophagy
CSF: cerebrospinal fluid
CV: coefficient of variation
HCD: higher energy collisional dissociation
HR-PRM: high resolution parallel reaction monitoring
hsc70: heat shock cognate 71 kDa protein
IP-HR-PRM-MS: hybrid immunoprecipitation high resolution parallel reaction monitoring mass spectrometry
IP: immunoprecipitation
MS: mass spectrometry
LC: liquid chromatography
LAMP2: lysosome-associated membrane glycoprotein 2
NCE: normalized collision energy
PBS: phosphate buffered saline
P-tau: phosphorylated tau protein
P-tau_181_: phosphorylated tau protein with threonine phosphorylation at position 181
PBS-T: PBS Tween
SRM: selected reaction monitoring
MS/MS: tandem mass spectrometry
T-tau: total tau protein
QC: quality control
