# Supplementary material for: Targeting LAMP2 in human cerebrospinal fluid with a combination of immunopurification and high resolution parallel reaction monitoring mass spectrometry
Source: Clin Proteomics. 2016 Feb 25;13:4. doi: 10.1186/s12014-016-9104-2 (PMC4768413; doi:10.1186/s12014-016-9104-2)
Supplement: Supplementary file 3 — 10.1186/s12014-016-9104-2 Supporting Information. Table legends for Tables S1–S3 and Supplementary Figures S1–S18. [file 12014_2016_9104_MOESM3_ESM.pdf]

ADDITIONAL FILE 3

SUPPORTING INFORMATION FOR:

Targeting LAMP2 in Human Cerebrospinal Fluid with a Combination of Immunopurification and High Resolution Parallel Reaction Monitoring Mass Spectrometry

AUTHORS

Simon Sjödin[†]*, Annika Öhrfelt[†], Gunnar Brinkmalm[†], Henrik Zetterberg[†,‡], Kaj Blennow[†] and Ann Brinkmalm[†]

[†]Institution of Neuroscience and Physiology, Department of Psychiatry and Neurochemistry, The Sahlgrenska Academy, University of Gothenburg, Sweden

[‡]UCL Institute of Neurology, University College London, United Kingdom

*To whom correspondence should be addressed: Simon Sjödin, Institute of Neuroscience and Physiology, Department of Psychiatry and Neurochemistry, The Sahlgrenska Academy at the University of Gothenburg, Sahlgrenska University Hospital at Mölndal, House V3, SE-431 80 Mölndal, Sweden, Tel.: +46-31-343-00-41, Fax: +46-31-41-92-89, E-mail: simon.sjodin@neuro.gu.se.

SUPPLEMENTARY TABLES

Table S1. Proteins identified by nano-LC-MS/MS. Human CSF was immunoprecipitated using an anti-LAMP2 or unspecific mouse serum antibody. The samples were dried by vacuum centrifugation, dissolved by using either 50 mM NH_4HCO_3 or 0.1 % RapiGest SF in 50 mM NH_4HCO_3 and digested using trypsin. The samples were prepared and analyzed in triplicates. The protein name, UniProtKB accession number, gene name, Mascot score, number of unique peptides and coverage are reported.

Table S2. Peptides identified by nano-LC-MS/MS. Human CSF was immunoprecipitated using an anti-LAMP2 antibody, dried by vacuum centrifugation, dissolved by using either 50 mM NH_4HCO_3 or 0.1 % RapiGest SF in 50 mM NH_4HCO_3 and digested using trypsin. The samples were prepared and analyzed in triplicates. The table reports the sequence, the amino acid sequence position (aa), number of charges, number of missed cleavages, monoisotopic mass, modifications, Mascot ion score, Mascot expect value and mass deviation (ΔM (ppm)).

Table S3. Quantitative analyses of HR-PRM-MS data. The selected ion fragments peak areas and ratio between the sums of the selected ion fragments peak areas of the tryptic peptide against that of the added isotope labeled peptide are presented in the table. The fragment ions selected are shown in Table 2. The sheets represent the data which has been used for producing the results presented in each figure as indicated by the name of the sheets.

SUPPLEMENTARY FIGURES

Figure S1. Ion fragment mass spectrum of the LAMP2 peptide aa 46-53.

Figure S2. Ion fragment mass spectrum of the LAMP2 peptide aa 133-144.

Figure S3. Ion fragment mass spectrum of the LAMP2 peptide aa 145-152.

Figure S4. Ion fragment mass spectrum of the LAMP2 peptide aa 153-161.

Figure S5. Ion fragment mass spectrum of the LAMP2 peptide aa 281-289.

Figure S6. Ion fragment mass spectrum of the LAMP2 peptide aa 334-351.

Figure S7. Micro-LC HR-PRM-MS method analysis of isotope labeled peptides. Mixtures of the isotope labeled LAMP2 peptides A, aa 133-144; B, aa 145-152; C, aa 153-161; D, aa 334-351 and the BSA peptide aa 421-433 were made in three different mixtures (Batch 1-3). Using micro-LC HR-PRM-MS the batches were analyzed in replicates of ten where the samples were either injected from multiple vials or multiple times from a single vial. The sum of fragment ion peak areas was used as the measure for determining variation. The sum of peak areas were normalized against the average sum of peak areas of the ten replicates and thus the samples relative variation is shown in the bar graphs. Furthermore, the replicates are sorted, from left to right, in order of analysis.

Figure S8. Micro-LC HR-PRM-MS method analysis of a BSA isotope labeled peptide. A dilution of the isotope labeled bovine serum albumin peptide aa 421-433 was made in three different mixtures (Batch 1-3). The mixture also included the LAMP2 peptides aa 133-144, 145-152, 153-161 and 334-351. Using micro-LC HR-PRM-MS the batches were analyzed in replicates of ten where the samples were either injected from multiple vials or multiple times

from a single vial. The sum of fragment ion peak areas was used as the measure for determining variation. The sum of peak areas were normalized against the average sum of peak areas of the ten replicates and thus the samples relative variation is shown in the bar graphs. Furthermore, the replicates are sorted, from left to right, in order of analysis.

Figure S9. Hybrid immunoprecipitation HR-PRM-MS method variability monitored with a BSA peptide. Using two methodological workflows (Figure 2) the variability in the IP-HR-PRM-MS method targeting LAMP2 was evaluated. Workflow 1 and 2 each included samples prepared on three separate occasions, Batch 1-3, each including 8 + 8 technical replicates (eight for each workflow). The different batches were analyzed on a single occasion to minimize the influence of altering instrumental performance. The workflow enabled determination of intra- and interday coefficient's of variations (CVs), where the intraday variation was calculated for each batch and the interday variation calculated for the samples included in all three batches. The intra- and interday CVs for the workflows are shown for the BSA peptide aa 421-433. The bar graphs show the calculated ratio between the sum of the included ion fragment peak areas of the tryptic peptide against the sum of peak areas of the added isotope labeled peptide for each sample and within each batch sorted, from left to right, in order of analysis.

Figure S10. HR-PRM fragment ion mass spectrum of the LAMP2 peptide aa 133-144. A, Total ion chromatogram showing the ion current of the analyzed fragment ions, y5, y6, y7, y8 and y9, in a retention time window of four minutes. The added isotope labeled peptide fragment ions are indicated in red and the tryptic peptide fragment ions in black. B, Representative fragment ion mass spectrum.

Figure S11. HR-PRM fragment ion mass spectrum of the LAMP2 peptide aa 145-152. A, Total ion chromatogram showing the ion current of the analyzed fragment ions, y4, y5, y6 and y7, in a retention time window of four minutes. The added isotope labeled peptide fragment ions are indicated in red and the tryptic peptide fragment ions in black. B, Representative fragment ion mass spectrum.

Figure S12. HR-PRM fragment ion mass spectrum of the LAMP2 peptide aa 153-161. A, Total ion chromatogram showing the ion current of the analyzed fragment ions, y4, y5, y6 and y7, in a retention time window of four minutes. The added isotope labeled peptide fragment ions are indicated in red and the tryptic peptide fragment ions in black. B, Representative fragment ion mass spectrum.

Figure S13. HR-PRM fragment ion mass spectrum of the LAMP2 peptide aa 334-351. A, Total ion chromatogram showing the ion current of the analyzed fragment ions, y6, y7, y8, y9, y10, y11, y12, y13 and y14, in a retention time window of four minutes. The added isotope labeled peptide fragment ions are indicated in red and the tryptic peptide fragment ions in black. B, Representative fragment ion mass spectrum.

Figure S14. HR-PRM fragment ion mass spectrum of the BSA peptide aa 421-433. Full length BSA and an isotope labeled peptide, aa 421-433, were added to samples immunoprecipitated against LAMP2. A, Total ion chromatogram showing the ion current of the analyzed fragment ions, y4, y6, y7, y8, y9 and y10, in a retention time window of four minutes. The added isotope labeled peptide fragment ions are indicated in red and the tryptic peptide fragment ions in black. B, Representative fragment ion mass spectrum.

Figure S15. Monitoring the hybrid immunoprecipitation HR-PRM-MS method stability and variance. Ten replicate running controls were produced by performing immunoprecipitation of LAMP2 from a quality control CSF pool. The running controls were analyzed in a randomized integrated order with a set of 14 control and 14 Alzheimer's disease (AD) subject samples. Shown is the ratio of the included ion fragment peak areas of the tryptic peptide against the sum of peak areas of the added isotope labeled peptide and the coefficient of variation (CV) calculated for the ratio of the 10 running controls. The bar graph show the value for each replicate sample sorted, in regard to the peptides, from left to right in order of analysis.

Figure S16. Correlation between the measured levels of LAMP2 peptides. Using the developed IP-HR-PRM-MS method the level of the three peptides LAMP2 aa 133-144, 145-152 and 153-161 was measured in AD (n =14) and control subjects (n = 14). Shown is the calculated ratio between the sum of the included ion fragment peak areas of the tryptic peptide against the sum of peak areas of the added isotope labeled peptide for the three peptides. Using Spearman's test of correlation the relationship between the combinations of the three peptide measurements was evaluated in AD (A, B and C, respectively) and in control subjects (D, E and F, respectively). Shown in each graph is the Spearman's ρ value and the p-value with a significance designated as p being < 0.01.

Figure S17. Correlation of LAMP2 peptides with core biomarkers in AD subjects. Using the developed IP-HR-PRM-MS method the level of the three LAMP2 peptides aa 133-144 (A-C), aa 145-152 (D-F) and aa 153-161 (G-I) was measured in subjects with an AD core biomarker profile (n = 14). Shown is the calculated ratio between the sum of the included ion fragment peak areas of the tryptic peptide against the sum of peak areas of the added isotope labeled peptide for the three peptides. The correlation between the three peptides and the core biomarkers was

determined using Spearman’s test of correlation. Shown in each graph is Spearman’s ρ value and the p-value with a significance designated as p being < 0.01.

Figure S18. Correlation of LAMP2 peptides with core biomarkers in control subjects. Using the developed IP-HR-PRM-MS method the level of the three LAMP2 peptides aa 133-144 (A-C), aa 145-152 (D-F) and aa 153-161 (G-I) was measured control subjects characterized by having a control core biomarker profile (n = 14). Shown is the calculated ratio between the sum of the included ion fragment peak areas of the tryptic peptide against the sum of peak areas of the added isotope labeled peptide for the three peptides. The correlation between the three peptides and the core biomarkers was determined using Spearman’s test of correlation. Shown in each graph is Spearman’s ρ value and the p-value with a significance designated as p being < 0.01.

Figure S1.

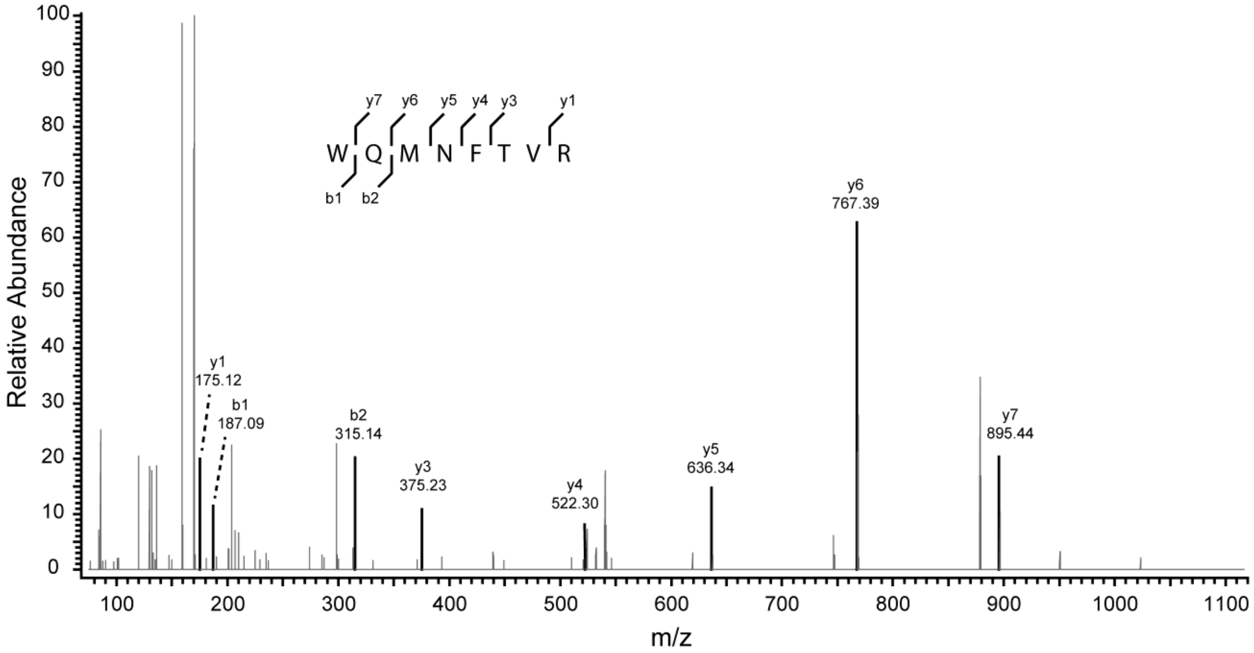

100
90
80
70
60
50
40
30
20
10
0
Relative Abundance
y7 y6 y5 y4 y3 y1
W Q M N F T V R
b1 b2
y6
767.39
y1
175.12
b1
187.09
b2
315.14
y3
375.23
y4
522.30
y5
636.34
y7
895.44
100 200 300 400 500 600 700 800 900 1000 1100
m/z

Figure S2.

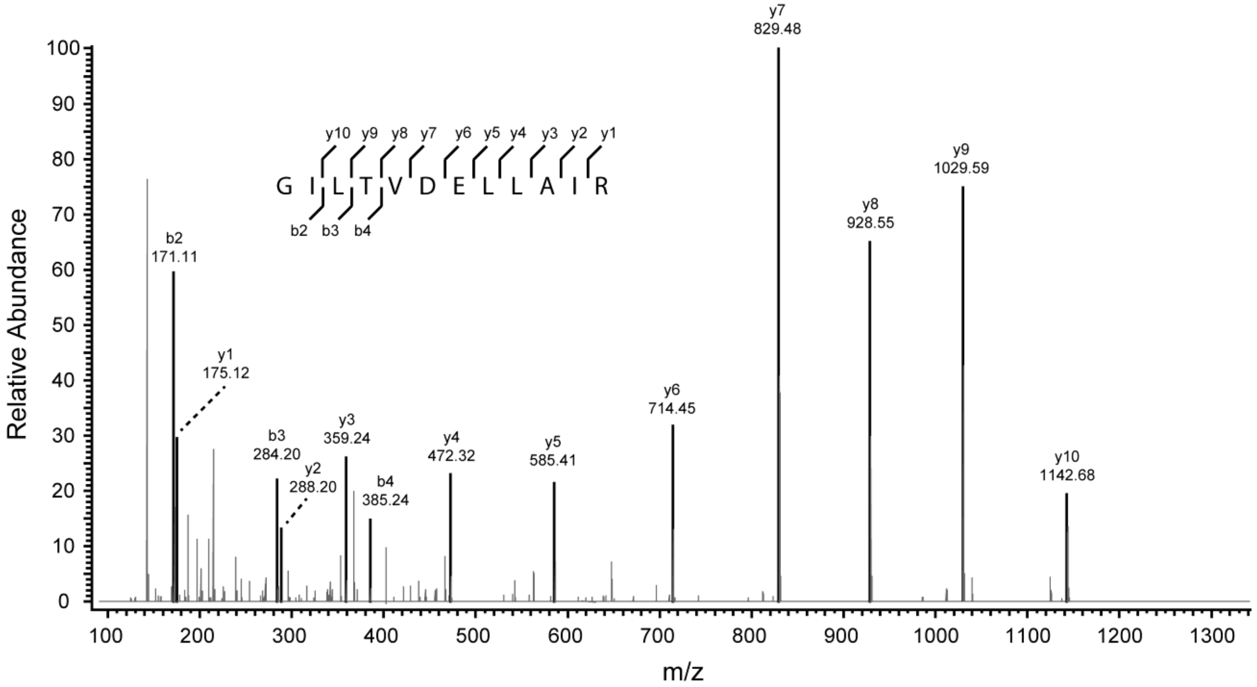

y10 y9 y8 y7 y6 y5 y4 y3 y2 y1
G I L T V D E L L A I R
b2 b3 b4
Relative Abundance
m/z
b2
171.11
y1
175.12
b3
284.20
y2
288.20
y3
359.24
b4
385.24
y4
472.32
y5
585.41
y6
714.45
y7
829.48
y8
928.55
y9
1029.59
y10
1142.68

Figure S3.

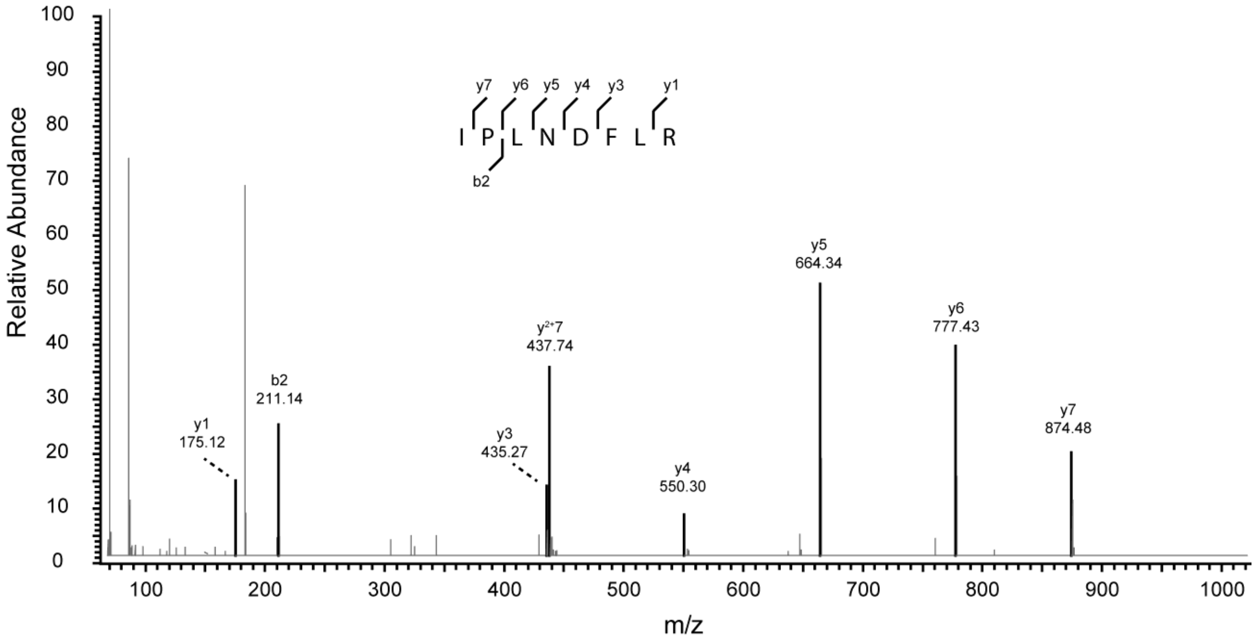

100
90
80
70
60
50
40
30
20
10
0
Relative Abundance
y7 y6 y5 y4 y3 y1
I P L N D F L R
b2
y5
664.34
y6
777.43
y²⁺7
437.74
b2
211.14
y7
874.48
y1
175.12
y3
435.27
y4
550.30
100
200
300
400
500
600
700
800
900
1000
m/z

Figure S4.

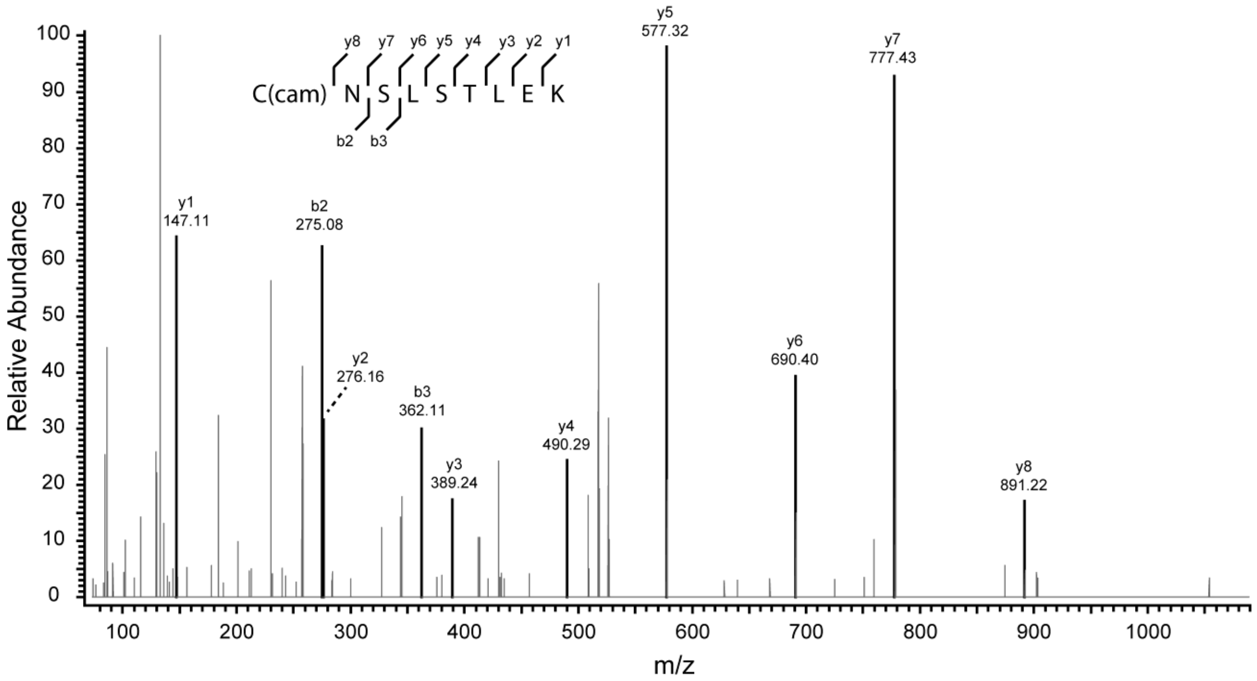
C(cam) N S L S T L E K
y8 y7 y6 y5 y4 y3 y2 y1
b2 b3
y1 147.11
b2 275.08
y2 276.16
b3 362.11
y3 389.24
y4 490.29
y5 577.32
y6 690.40
y7 777.43
y8 891.22
Relative Abundance
m/z

Figure S5.

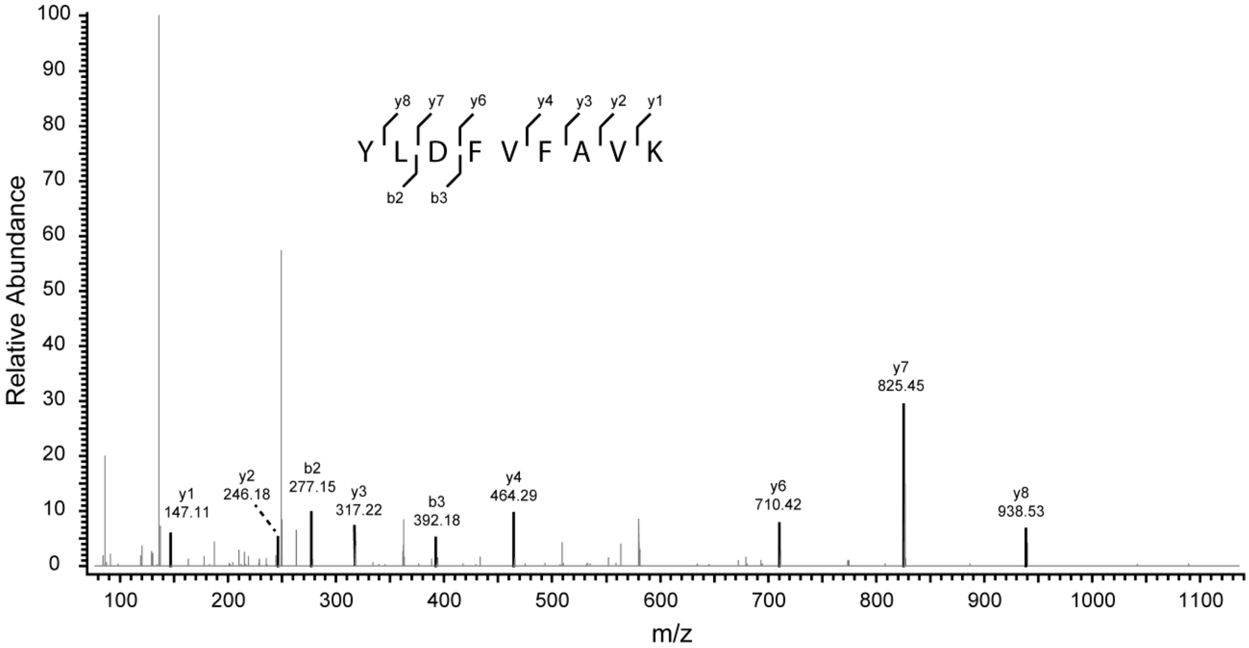

YLDFVFAVK
y8
y7
y6
y4
y3
y2
y1
b2
b3
y1
147.11
y2
246.18
b2
277.15
y3
317.22
b3
392.18
y4
464.29
y6
710.42
y7
825.45
y8
938.53
Relative Abundance
m/z

Figure S6.

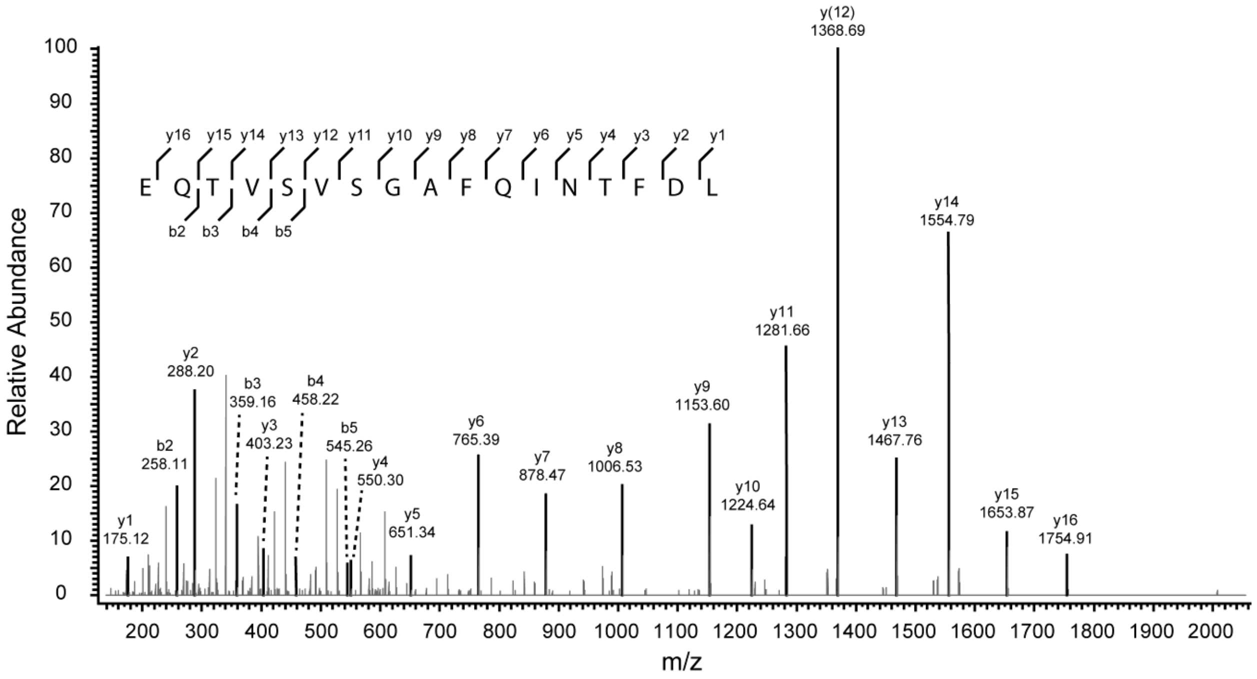

y16 y15 y14 y13 y12 y11 y10 y9 y8 y7 y6 y5 y4 y3 y2 y1
E Q T V S V S G A F Q I N T F D L
b2 b3 b4 b5
Relative Abundance
m/z
y1 175.12
b2 258.11
y2 288.20
b3 359.16
y3 403.23
b4 458.22
b5 545.26
y4 550.30
y5 651.34
y6 765.39
y7 878.47
y8 1006.53
y9 1153.60
y10 1224.64
y11 1281.66
y(12) 1368.69
y13 1467.76
y14 1554.79
y15 1653.87
y16 1754.91

Figure S7.

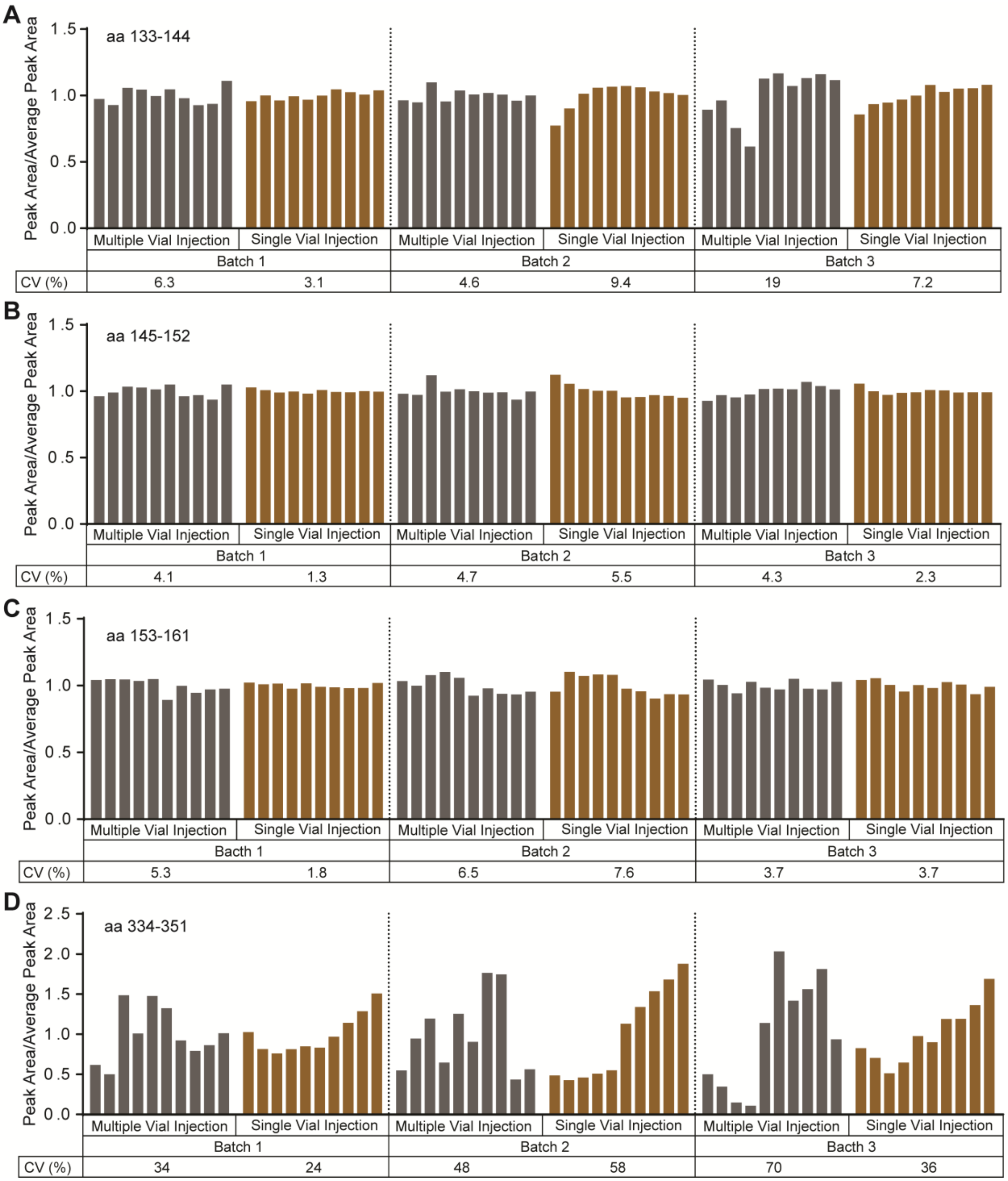
A
Peak Area/Average Peak Area
aa 133-144
Multiple Vial Injection
Single Vial Injection
Batch 1
Batch 2
Batch 3
CV (%)
6.3
3.1
4.6
9.4
19
7.2
B
aa 145-152
CV (%)
4.1
1.3
4.7
5.5
4.3
2.3
C
aa 153-161
Bacth 1
CV (%)
5.3
1.8
6.5
7.6
3.7
3.7
D
aa 334-351
Bacth 3
CV (%)
34
24
48
58
70
36

Figure S8.

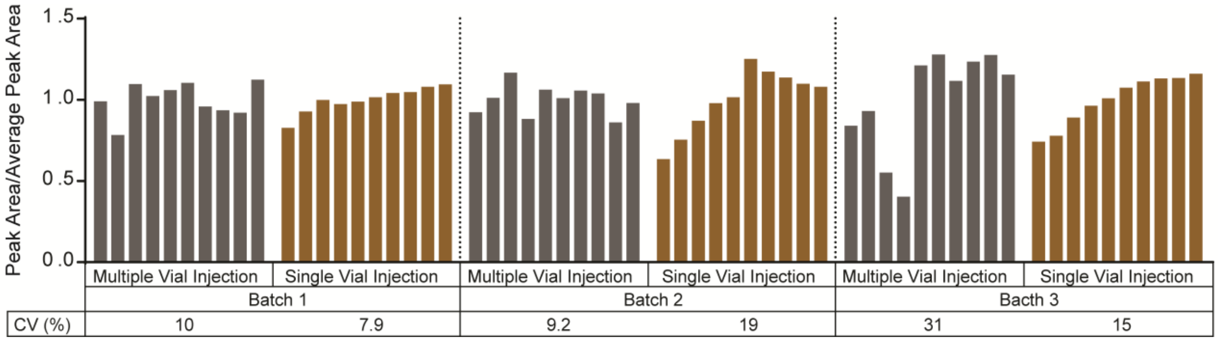
Peak Area/Average Peak Area
1.5
1.0
0.5
0.0
Multiple Vial Injection
Single Vial Injection
Multiple Vial Injection
Single Vial Injection
Multiple Vial Injection
Single Vial Injection
Batch 1
Batch 2
Bacth 3
CV (%)
10
7.9
9.2
19
31
15

Figure S9.

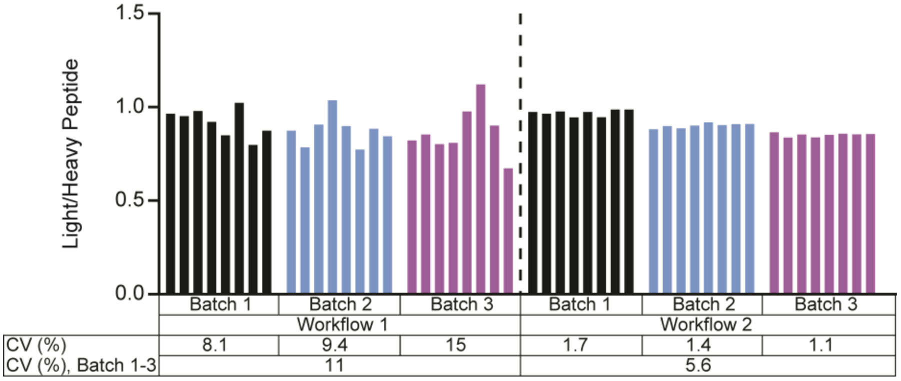

	Workflow 1 Batch 1	Workflow 1 Batch 2	Workflow 1 Batch 3	Workflow 2 Batch 1	Workflow 2 Batch 2	Workflow 2 Batch 3
CV (%)	8.1	9.4	15	1.7	1.4	1.1
CV (%), Batch 1-3	11			5.6		

Figure S10.

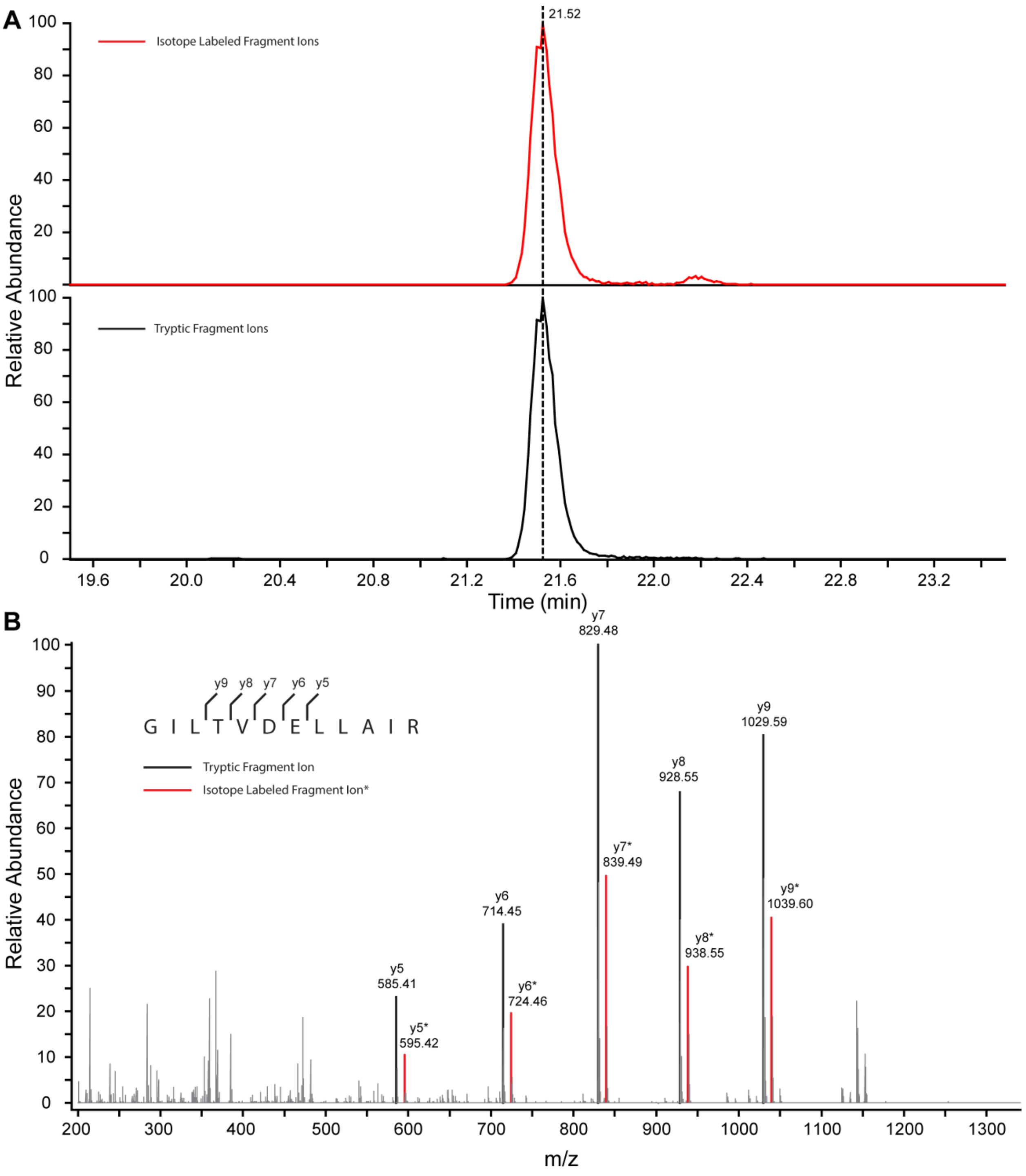

A
21.52
Isotope Labeled Fragment Ions
Tryptic Fragment Ions
Relative Abundance
Time (min)
B
GILTVDELLAIR
y9 y8 y7 y6 y5
Tryptic Fragment Ion
Isotope Labeled Fragment Ion*
y5 585.41
y5* 595.42
y6 714.45
y6* 724.46
y7 829.48
y7* 839.49
y8 928.55
y8* 938.55
y9 1029.59
y9* 1039.60
Relative Abundance
m/z

Figure S11.

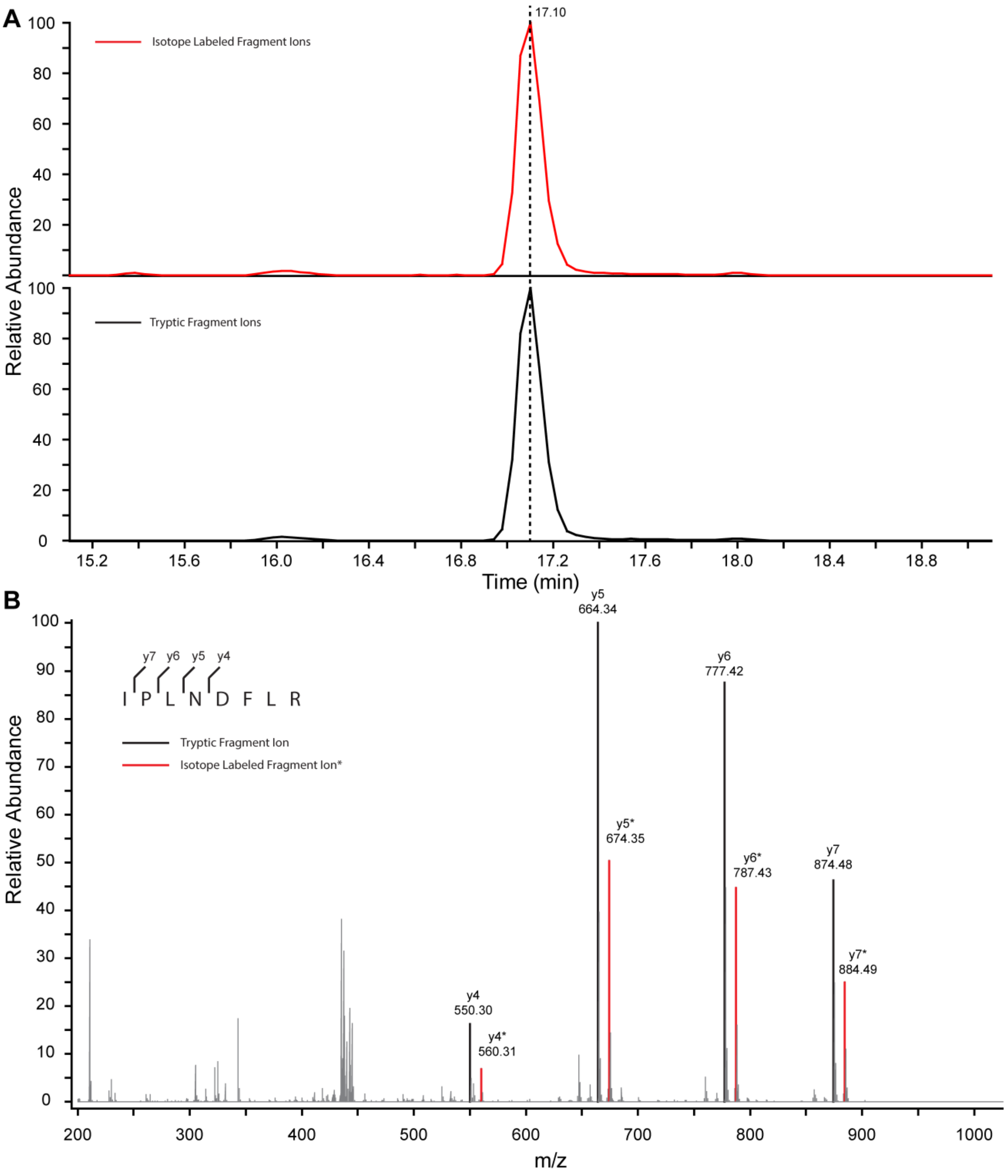
A
17.10
Isotope Labeled Fragment Ions
Tryptic Fragment Ions
Relative Abundance
Time (min)
B
y7 y6 y5 y4
I P L N D F L R
Tryptic Fragment Ion
Isotope Labeled Fragment Ion*
y5
664.34
y6
777.42
y5*
674.35
y6*
787.43
y7
874.48
y7*
884.49
y4
550.30
y4*
560.31
Relative Abundance
m/z

Figure S12.

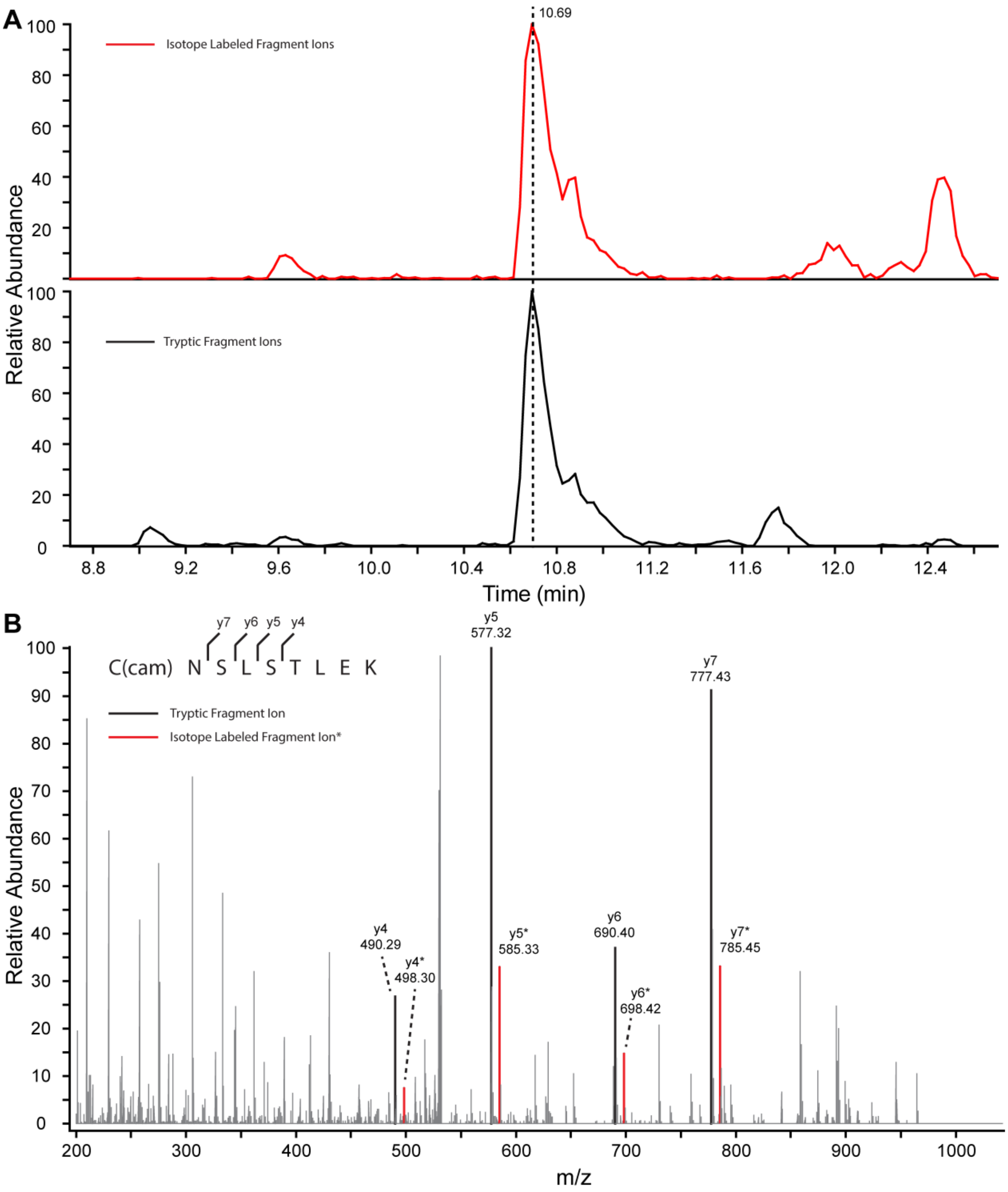

A
10.69
Isotope Labeled Fragment Ions
Tryptic Fragment Ions
Relative Abundance
Time (min)
B
C(cam) N S L S T L E K
y7 y6 y5 y4
Tryptic Fragment Ion
Isotope Labeled Fragment Ion*
y5
577.32
y7
777.43
y4
490.29
y4*
498.30
y5*
585.33
y6
690.40
y6*
698.42
y7*
785.45
Relative Abundance
m/z

Figure S13.

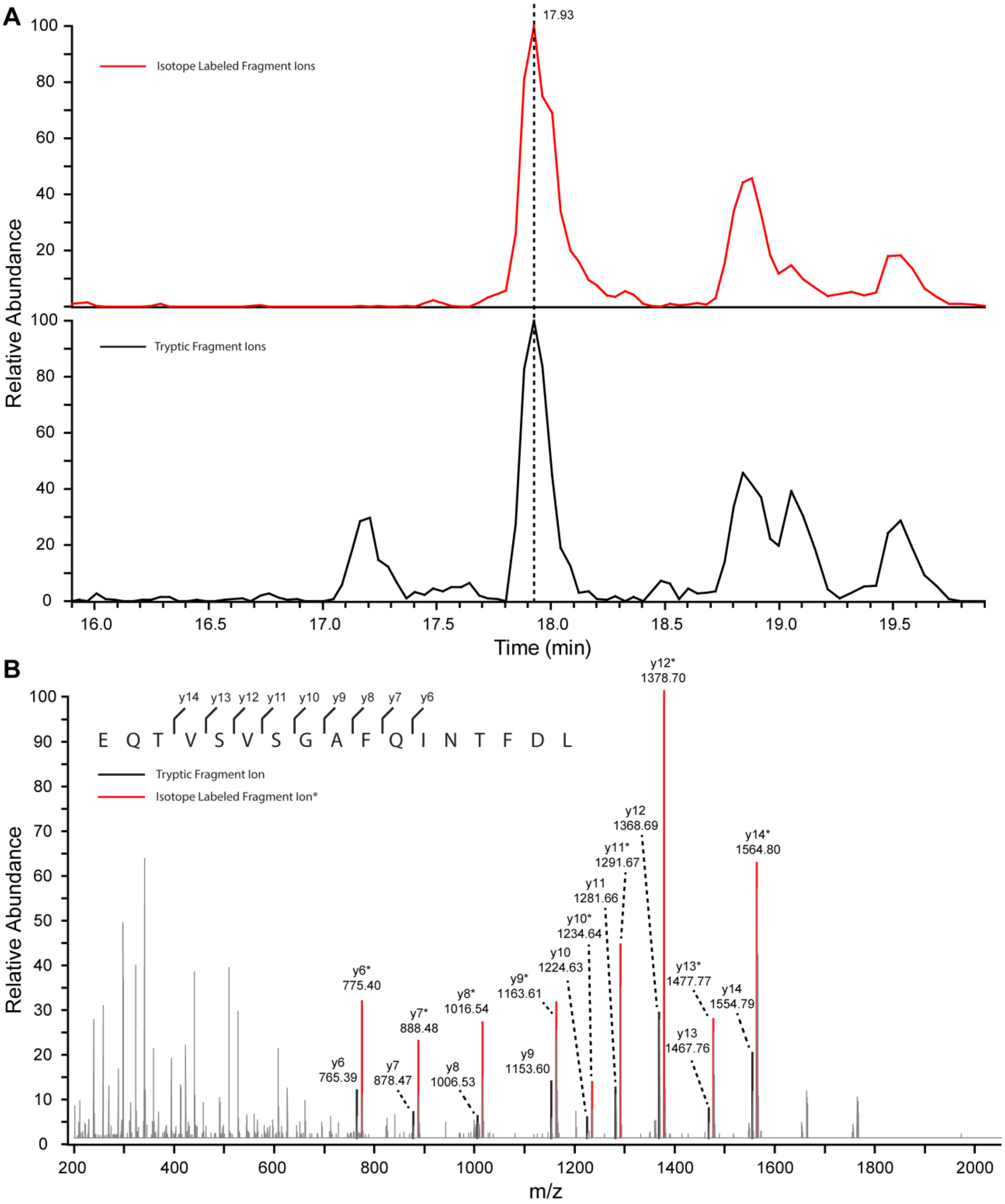
A
17.93
Isotope Labeled Fragment Ions
Tryptic Fragment Ions
Relative Abundance
Time (min)
B
EQTVSVSGAFQINTFDL
y14 y13 y12 y11 y10 y9 y8 y7 y6
Tryptic Fragment Ion
Isotope Labeled Fragment Ion*
y12* 1378.70
y12 1368.69
y11* 1291.67
y11 1281.66
y10* 1234.64
y10 1224.63
y9* 1163.61
y9 1153.60
y8* 1016.54
y8 1006.53
y7* 888.48
y7 878.47
y6* 775.40
y6 765.39
y14* 1564.80
y14 1554.79
y13* 1477.77
y13 1467.76
Relative Abundance
m/z

Figure S14.

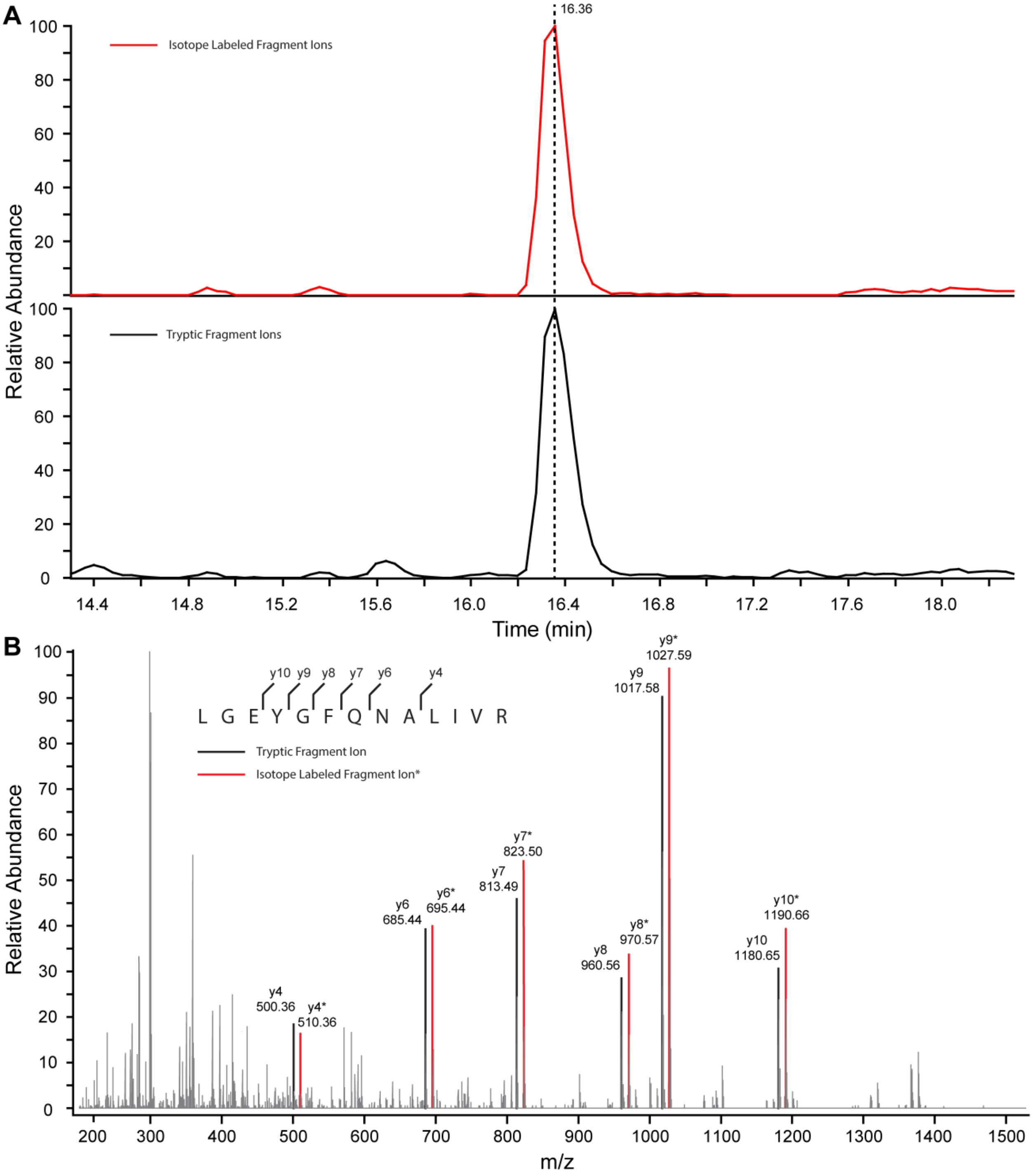
A
16.36
Isotope Labeled Fragment Ions
Tryptic Fragment Ions
Relative Abundance
Time (min)
B
y10 y9 y8 y7 y6 y4
L G E Y G F Q N A L I V R
Tryptic Fragment Ion
Isotope Labeled Fragment Ion*
y4 500.36
y4* 510.36
y6 685.44
y6* 695.44
y7 813.49
y7* 823.50
y8 960.56
y8* 970.57
y9 1017.58
y9* 1027.59
y10 1180.65
y10* 1190.66
Relative Abundance
m/z

Figure S15.

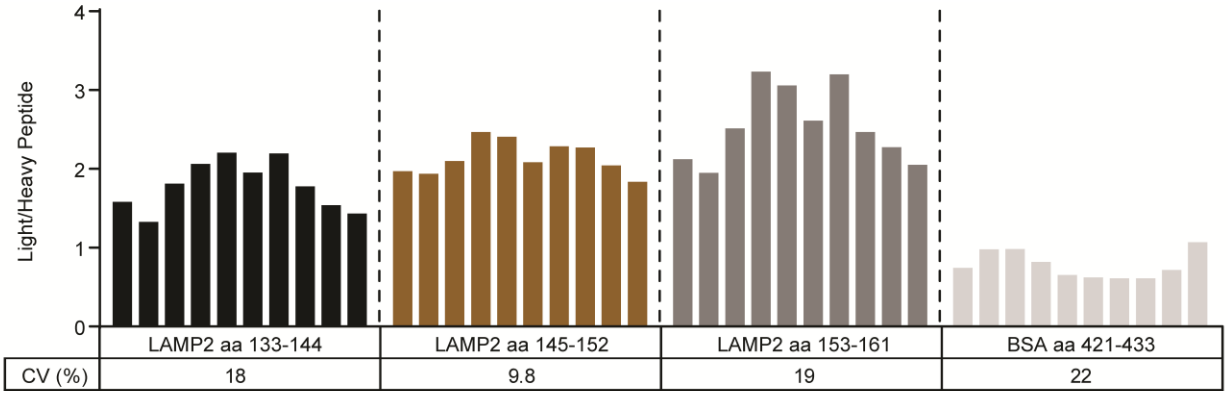
Light/Heavy Peptide
0
1
2
3
4
LAMP2 aa 133-144
LAMP2 aa 145-152
LAMP2 aa 153-161
BSA aa 421-433
CV (%)
18
9.8
19
22

Figure S16.

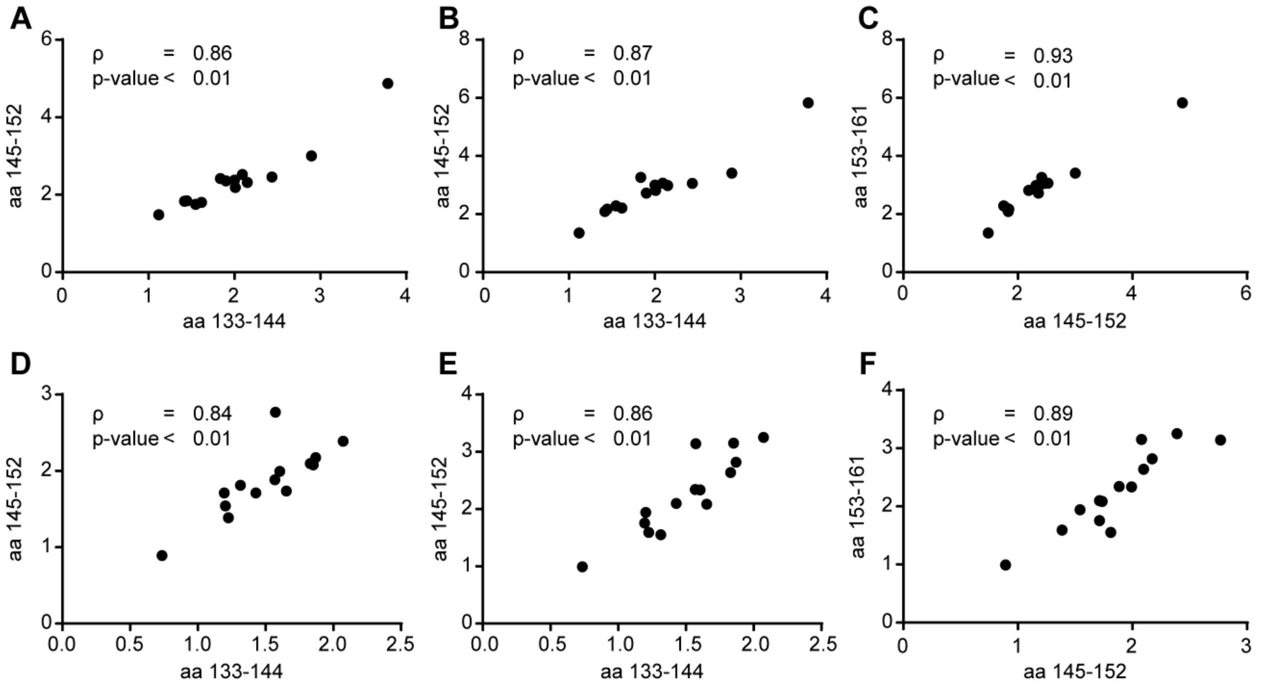

A
ρ = 0.86
p-value < 0.01
aa 145-152
aa 133-144
B
ρ = 0.87
p-value < 0.01
aa 145-152
aa 133-144
C
ρ = 0.93
p-value < 0.01
aa 153-161
aa 145-152
D
ρ = 0.84
p-value < 0.01
aa 145-152
aa 133-144
E
ρ = 0.86
p-value < 0.01
aa 145-152
aa 133-144
F
ρ = 0.89
p-value < 0.01
aa 153-161
aa 145-152

Figure S17.

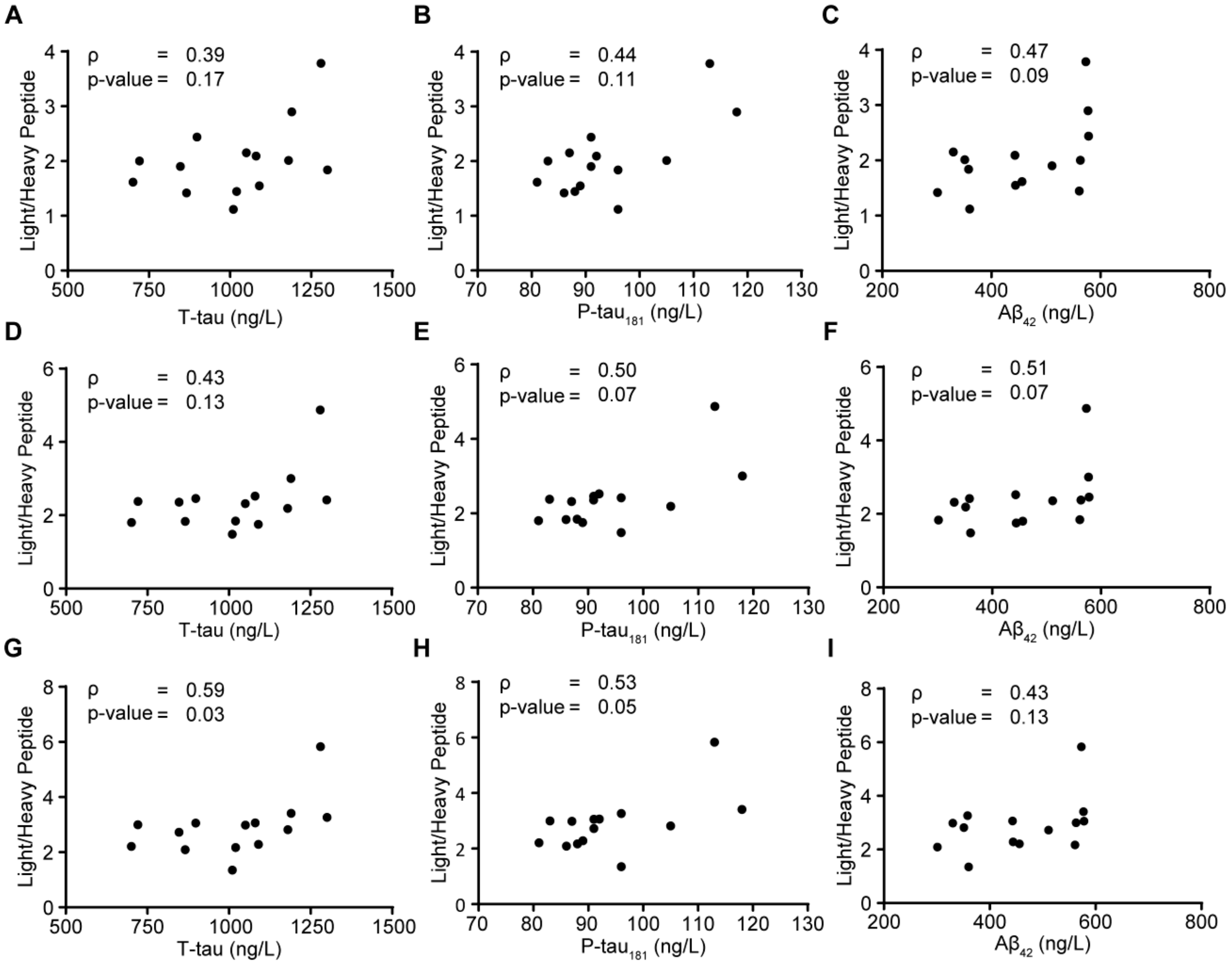
A
ρ = 0.39
p-value = 0.17
Light/Heavy Peptide
T-tau (ng/L)
B
ρ = 0.44
p-value = 0.11
Light/Heavy Peptide
P-tau181 (ng/L)
C
ρ = 0.47
p-value = 0.09
Light/Heavy Peptide
Aβ42 (ng/L)
D
ρ = 0.43
p-value = 0.13
Light/Heavy Peptide
T-tau (ng/L)
E
ρ = 0.50
p-value = 0.07
Light/Heavy Peptide
P-tau181 (ng/L)
F
ρ = 0.51
p-value = 0.07
Light/Heavy Peptide
Aβ42 (ng/L)
G
ρ = 0.59
p-value = 0.03
Light/Heavy Peptide
T-tau (ng/L)
H
ρ = 0.53
p-value = 0.05
Light/Heavy Peptide
P-tau181 (ng/L)
I
ρ = 0.43
p-value = 0.13
Light/Heavy Peptide
Aβ42 (ng/L)

Figure S18.

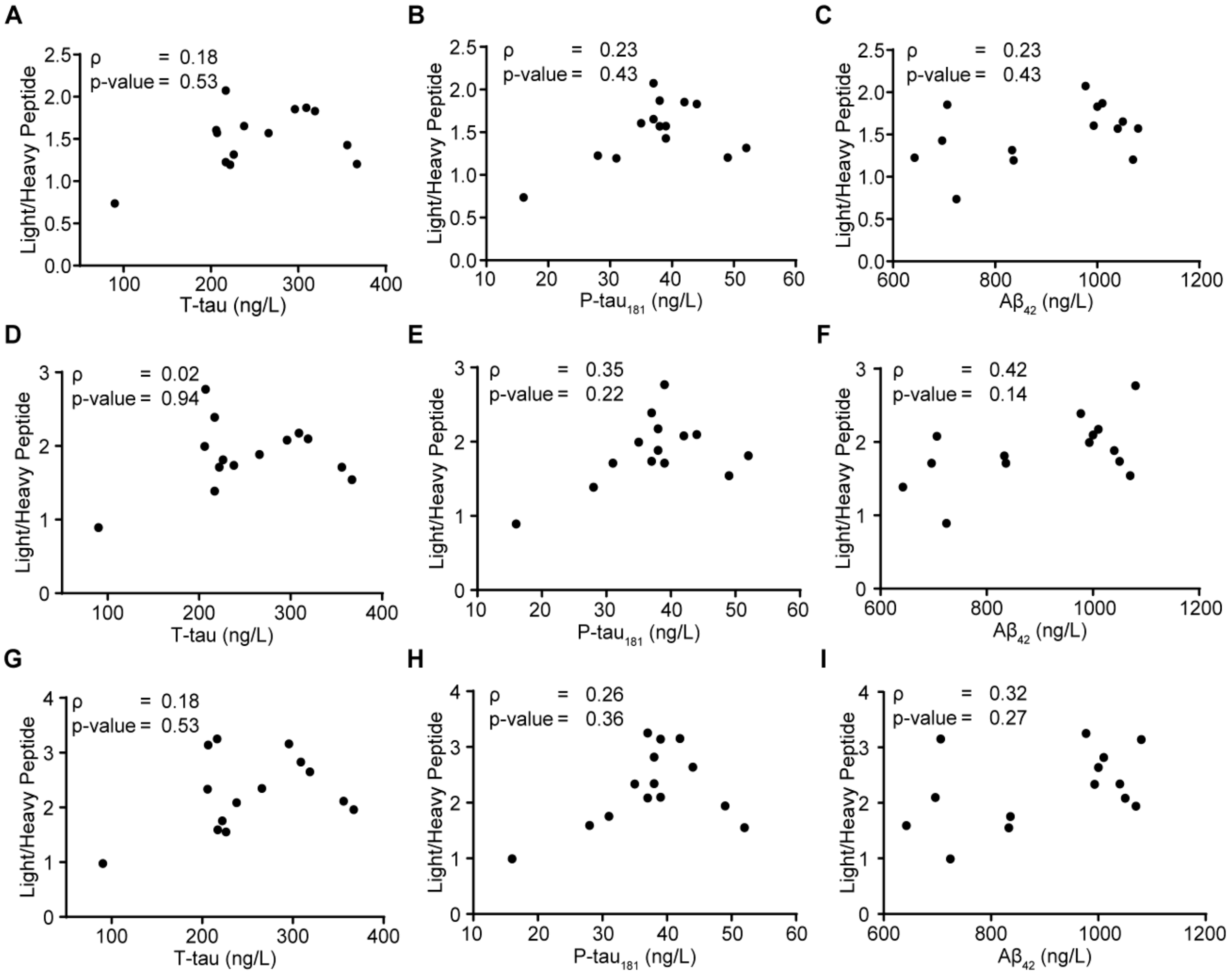
A
ρ = 0.18
p-value = 0.53
Light/Heavy Peptide
T-tau (ng/L)
B
ρ = 0.23
p-value = 0.43
Light/Heavy Peptide
P-tau181 (ng/L)
C
ρ = 0.23
p-value = 0.43
Light/Heavy Peptide
Aβ42 (ng/L)
D
ρ = 0.02
p-value = 0.94
Light/Heavy Peptide
T-tau (ng/L)
E
ρ = 0.35
p-value = 0.22
Light/Heavy Peptide
P-tau181 (ng/L)
F
ρ = 0.42
p-value = 0.14
Light/Heavy Peptide
Aβ42 (ng/L)
G
ρ = 0.18
p-value = 0.53
Light/Heavy Peptide
T-tau (ng/L)
H
ρ = 0.26
p-value = 0.36
Light/Heavy Peptide
P-tau181 (ng/L)
I
ρ = 0.32
p-value = 0.27
Light/Heavy Peptide
Aβ42 (ng/L)
